# Autophagy Activation Associates with Suppression of Prion Protein and Improved Mitochondrial Status in Glioblastoma Cells

**DOI:** 10.3390/cells12020221

**Published:** 2023-01-04

**Authors:** Paola Lenzi, Carla L. Busceti, Gloria Lazzeri, Rosangela Ferese, Francesca Biagioni, Alessandra Salvetti, Elena Pompili, Valerio De Franchis, Stefano Puglisi-Allegra, Alessandro Frati, Michela Ferrucci, Francesco Fornai

**Affiliations:** 1 Department of Translational Research and New Technologies in Medicine and Surgery, University of Pisa, Via Roma 55, 56126 Pisa, Italy; 2 Istituto di Ricovero e Cura a Carattere Scientifico (I.R.C.C.S.) Neuromed, Via Atinense 18, 86077 Pozzilli, Italy; 3 Department of Clinical and Experimental Medicine, University of Pisa, Via Roma 55, 56126 Pisa, Italy; 4 Department of Anatomy, Histology, Forensic Medicine and Orthopedics, Sapienza University of Rome, Via A. Borelli 50, 00161 Rome, Italy; 5 Neurosurgery Division, Department of Human Neurosciences, Sapienza University, 00135 Roma, Italy

**Keywords:** mTOR, cytofluorimetry, mitochondrial fission, Pink1, Parkin, Fis1, DRP1 lysosomes, rapamycin

## Abstract

Cells from glioblastoma multiforme (GBM) feature up-regulation of the mechanistic Target of Rapamycin (mTOR), which brings deleterious effects on malignancy and disease course. At the cellular level, up-regulation of mTOR affects a number of downstream pathways and suppresses autophagy, which is relevant for the neurobiology of GBM. In fact, autophagy acts on several targets, such as protein clearance and mitochondrial status, which are key in promoting the malignancy GBM. A defective protein clearance extends to cellular prion protein (PrPc). Recent evidence indicates that PrPc promotes stemness and alters mitochondrial turnover. Therefore, the present study measures whether in GBM cells abnormal amount of PrPc and mitochondrial alterations are concomitant in baseline conditions and whether they are reverted by mTOR inhibition. Proteins related to mitochondrial turnover were concomitantly assessed. High amounts of PrPc and altered mitochondria were both mitigated dose-dependently by the mTOR inhibitor rapamycin, which produced a persistent activation of the autophagy flux and shifted proliferating cells from S to G1 cell cycle phase. Similarly, mTOR suppression produces a long-lasting increase of proteins promoting mitochondrial turnover, including Pink1/Parkin. These findings provide novel evidence about the role of autophagy in the neurobiology of GBM.

## 1. Introduction

Up-regulation of the mechanistic Target of Rapamycin (mTOR) in glioblastoma multiforme (GBM) is established in human patients and experimental models [1,2,3,4,5]. An excess of mTOR activity leads to detrimental effects by acting on a number of intracellular pathways [6,7,8,9,10]. Among these, suppression of autophagy and impairment of mitochondrial turn-over prevail at large [11]. In fact, the loss of autophagy activity correlates with disease severity, tumor relapse, and resistance to therapy, while it stimulates the shift towards a stem cell phenotype. All these effects are responsible for a poor prognosis in GBM patients. In contrast, when autophagy is re-established via mTOR inhibition, an improvement of mitochondrial status and differentiated cell phenotype occur in GBM cells [6,7,12]. Thus, suppression of the autophagy pathway is sufficient to produce cell pathology induced by mTOR overexpression. In fact, when autophagy is stimulated cell pathology is greatly reduced [6]. Despite its prominent role, autophagy suppression implicates a number of downstream phenomena. Thus, the role played by autophagy in the biology of GBM cells is multifaceted. The role of autophagy in the clearance of misfolded proteins may extend to clear the cells from proteins which are relevant for cancerogenesis. In fact, in previous manuscripts we described that autophagy promotes the suppression of cell proliferation [6], it occludes the expression of stem cell markers such as nestin [7] and clears GBM cells from misfolded proteins [13,14].

A bulk of proteins owing influence on neighboring or distant cells may be overproduced or under-metabolized during autophagy suppression. This is the case of the cellular prion protein (PrPc). In line with this, recent evidence indicates that PrPc plays a key role in promoting GBM malignancy [15,16]. It is remarkable that, in prion disorders, elevated PrPc was shown to induce a defect in mitochondrial removal. This is likely to be caused by PrPc-mediated suppression of PTEN-induced putative kinase1 (Pink1)/Parkin (PARK) proteins at mitochondrial level [17]. Thus, one may hypothesize that an impairment of the autophagy machinery may induce an increase in PrPc [18], which in turn suppresses mitochondria turn-over, thus altering the mitochondrial compartment. This would lead to a vicious circle, since mitochondrial aberrations promote cell pathology in GBM [12,19].

Therefore, the present research study is designed to assess whether activation of the autophagy flux following mTOR inhibition leads to a significant clearance of PrPc along with removal of altered mitochondria concomitantly with persistent inhibition of cell proliferation.

In detail, the present study investigates whether PrPc accumulation along with mitochondrial impairment may all be reverted along with the expression of specific proteins promoting mitochondrial turn-over, when the autophagy flux is rescued through mTOR inhibition. The present study investigates whether all these effects, following a brief exposure to the mTOR inhibitor rapamycin, are long-lasting when administered to different GBM cell lines. This includes a persistent removal of PrPc and a long-lasting correction of the aberrant mitochondrial status.

## 2. Materials and Methods

### 2.1. Experimental Design

Experiments were carried out in human U87MG and A172 GBM cell lines. U87MG cells were obtained from Cell Bank (IRCCS San Martino-Institute, Genova, Italy). U87MG cells were maintained in DMEM growth medium (Sigma-Aldrich, Saint Louis, MO, USA) containing 10% Fetal Bovine Serum (FBS, Sigma-Aldrich, Saint Louis, MO, USA), 1% of MEM Non-Essential Amino-Acid (MEM-NEAA, Sigma-Aldrich, Saint Louis, MO, USA), penicillin, and streptomycin (50 IU/mL and 100 μg respectively, Sigma-Aldrich, Saint Louis, MO, USA). A172 cells were obtained from the European Collection of Authenticated Cell Cultures (ECACC) and from Cell Bank (IRCCS San Martino-IST, Genova) and they were maintained in Modified Eagle’s Medium (Euroclone, Milan, Italy) supplemented with 10% FBS, 2 mM L-glutamine, 100 IU/mL penicillin and 100 μg streptomycin. Both GBM cell lines were kept at 37 °C in a humidified atmosphere containing 5% CO_2_.

Based on previous studies [12,19], we selected a protocol where rapamycin is administered for a short time interval (24 h) and measurement of autophagy flux, cell cycle, analysis of the mitochondria status (mitochondrial alterations, mitophagy and fission), and the tumorigenic protein, i.e., prion protein, are carried out at various time intervals following rapamycin withdrawal, ranging from 1 day up to 14 days for morphology and 4 days up 14 days for autophagy flux and cell cycle. The timing and dosing of rapamycin (10 nM) were selected based on pilot experiments and previous studies [12,19]. In detail, the dose of rapamycin (10 nM) was selected according to the average of its therapeutic range (from 3 nM up to15 nM [20]). After rapamycin exposure (24 h), cell cultures were washed to remove rapamycin and kept in the culture medium, for 1 d, 4 d, 7 d, or 14 d. To keep cells alive for long time intervals (up to 14d), the culture medium was removed and replaced with fresh medium every three days.

The treatment solution of rapamycin (Sigma-Aldrich, Saint Louis, MO, USA) was prepared starting from a stock solution of 1 mM, which was dissolved in 1.41 M DMSO (Sigma-Aldrich, Saint Louis, MO, USA), and further diluted in the culture medium before being administered to the cell cultures. In this way, the final concentration of DMSO was 0.01%. Control cells were grown in the same culture medium containing 0.01% DMSO for the same time intervals and with the same washing procedure.

In experiments aimed at assessing the autophagy flux, the autophagy inhibitor bafilomycin A1 (100 nM, Sigma-Aldrich, Saint Louis, MO, USA) was added to the culture medium 3 h before the end of the treatments.

### 2.2. Light Microscopy for Concomitant MitoTracker Green and Immunohistochemistry for LC3 or BNIP3

To label mitochondria in living cells, MitoTracker-Green (MTR-G) (Thermo-Fisher Scientific, Waltham, MA, USA) dye was used, which labels total mitochondria [21,22]. Briefly, 5 × 10^4^ GBM cells were grown in 24-well plates containing 1 mL/well of culture medium. At the end of each experiment, the medium was removed, and cells were incubated in a serum free culture medium containing MTR-G at 500 nM for 45 min, at 37 °C and 5% CO_2_. At the end of incubation, MTR-G solution was removed, and GBM cells were washed in fresh pre-warmed medium before being fixed in 4% paraformaldehyde for 10 min. After fixation, GBM cells were permeabilized with 0.1% Triton-X 100 (Sigma-Aldrich, Saint Louis, MO, USA) for 15 min, and then incubated in 10% normal goat serum in PBS at 21 °C for 1h, followed by incubation with primary antibodies. In detail, microtubule-associated protein I/II light chain 3 (LC3, Abcam, Cambridge, UK) or BCL2/adenovirus E1B interacting protein 3 (BNIP3, Thermo-Fisher Scientific, Waltham, MA, USA) primary antibodies were used. GBM cells were incubated with rabbit anti-LC3 (diluted 1:75) or rabbit anti-BNIP3 (diluted 1:50), at 4 °C overnight. Primary antibodies’ solutions were prepared in PBS containing 2% normal goat serum. After washing in PBS, cells were incubated at 21 °C in the dark for 90 min with fluorophore-conjugated secondary antibody Alexa Fluor 546 (anti-rabbit, 1:200, Life Technologies, Carlsabad, CA, USA) in PBS. Cells were observed under light microscope Nikon Eclipse 80i (Nikon, Tokyo, Japan) equipped with a fluorescent lamp and a digital camera connected to NIS Element Software for image analysis (Nikon, Tokyo, Japan). Negative control cells were incubated with secondary antibodies only. Stained pictures were acquired independently and then they were merged. The number of double MTR-G + LC3 or MTR-G+BNIP3 puncta per cell was counted. Values are given as the mean number ± SEM per cell from N = 100 cells/group. Optical density of BNIP3 fluorescent cells was measured using Image J software (NIH, Version 1.8.0_172, Bethesda, MD, USA). Values are given as the mean percentage ± SEM from N = 100 cells/group.

### 2.3. Immunohistochemistry

GBM cells (N = 5 × 10^4^) were grown on poly-lysine slides placed in 24-well plates containing 1 mL/well of culture medium. At the end of the treatments, cells were washed in PBS and fixed with 4% paraformaldehyde in PBS for 15 min, incubated in 0.1% TritonX-100 for 15 min in PBS, and then blocked in PBS + 10% normal goat serum for 1h at 21 °C. Cells were then incubated overnight at 4 °C in 1% normal goat serum in PBS containing the primary antibodies (diluted 1:50) according to the following combinations: (i) anti-Phospho-S6 Ribosomal Protein (PS6RP, Cell Signaling, Milan, Italy); (ii) anti-Phospho p70 S6 Kinase (P70S6K) (Cell Signaling); (iii) anti-LC3 (Abcam) + Cathepsin D (Cat D) (Sigma-Aldrich, Saint Louis, MO, USA), (iv) anti-Pink1 (Abcam) + anti-PARK (Millipore, Burlington, MA, 808 USA); (v) anti-mitochondrial fission 1 protein (Fis1, GeneTex, Irvine, CA, USA) + anti-dynamin-related protein 1 (DRP1, Abcam) antibodies. After rinsing in PBS, GBM cells were incubated at 21 °C for 1h with the appropriate fluorophore-conjugated secondary antibodies (i.e., Alexa 488, Life Technologies, or Alexa 594 Life Technologies, Carlsabad, CA, USA) diluted 1:200. After washing in PBS, cells were transferred on coverslip and were mounted with the mounting medium Fluoroshield (Sigma-Aldrich, Saint Louis, MO, USA). GBM cells were observed under the Nikon Eclipse 80i light microscope (Nikon) equipped with a fluorescent lamp and a digital camera connected to the NIS Elements Software for image analysis (Nikon). Negative control cells were incubated with secondary antibodies only. Double stained pictures were acquired independently and then they were merged. Single fluorescent pictures were used to measure the optical density, using Image J software (NIH, Version 1.8.0_172, Version 1.8.0_172, Bethesda, MD, USA, Version 1.8.0_172). Values are given as the mean percentage ± SEM from N = 100 cells/group. Merged pictures were used to count the number of double fluorescent puncta per cell. Values are given as the mean number ± SEM per cell from N = 100 cells/group.

### 2.4. Confocal Microscopy

For confocal microscopy, 5 × 10^2^ GBM cells were grown on poly-lysine slides placed in 24-well plates containing 1 mL/well of culture medium.

Cells were washed in PBS and fixed with methanol at 21 °C for 5 min, followed by incubation in 100 mM Tris-HCl, 5% urea at 95 °C for 10 min. Then, GBM cells were permeabilized in 0.2% Triton X-100 for 10 min and were blocked in PBS containing 0.1% Tween-20 (PBST), 1% bovine serum albumin (BSA), and 22.52 mg/mL of glycine for 30 min. Cells were then incubated in the primary antibodies’ solution overnight at 4 °C. Primary antibodies were diluted 1:50 in in PBST with 1% BSA. After washing in PBST, GBM cells were incubated at 21 °C for 1h with the appropriate fluorophore-conjugated secondary antibodies (i.e., Alexa 488, Life Technologies, or Alexa 594 Life Technologies, Carlsabad, CA, USA) diluted 1:200 in PBST with 1% BSA. After incubation with secondary antibody, GBM cells were washed in PBS, mounted in Prolong Diamond Antifade Mountant (Life Technologies, Carlsabad, CA, USA) and observed under a Leica TCSSP5 confocal laser-scanning microscope (Leica Microsystems, Wetzlar, Germany) using a sequential scan procedure. Confocal images were collected every 400 nm intervals through the z-axis by means of 63-oil lenses. Z-stacks of serial optical planes were analyzed using the Multicolor Packages (Leica Microsystems, Wetzlar, Germany). Negative control GBM cells were incubated with secondary antibodies only. The optical density of each single fluorescent antigen was measured using Image J software (NIH, Bethesda, MD, USA, Version 1.8.0_172) and values are given as the mean percentage ± SEM from N = 100 cells/group. The number of double fluorescent puncta per cell was counted and values are given as the mean number ± SEM per cell from N = 100 cells/group.

### 2.5. Transmission Electron Microscopy (TEM)

GBM cells (1 × 10^6^) were seeded in 10 mm diameter culture dishes with 5 mL of culture medium. After removing culture medium, cells were fixed with the first fixing solution (2.0% paraformaldehyde/0.1% glutaraldehyde in 0.1M PBS pH 7.4) for 90 min at 4 °C. Then cells were gently scraped from the plate, centrifuged at 10,000× *g* rpm for 10 min and cell pellet was collected, washed in PBS, and fixed with the second fixing solution (1% osmium tetroxide) for 1 h at 4 °C. After washing, cell pellet was dehydrated in increasing ethanol solutions and embedded in epoxy resin.

Either plain electron microscopy or immuno-electron microscopy was carried out in ultra-thin sections, which were obtained at ultra-microtome (Leica Microsystems, Wetzlar, Germany) and were observed at Jeol JEM SX100 Transmission Electron Microscope (TEM, Jeol, Tokyo, Japan).

### 2.6. Ultra-Structural Analysis of Mitochondria

To analyze mitochondria status, non-serial ultra-thin sections (90 nm thick) were examined at TEM at 6000× magnification. Several grids were observed in order to analyse a total number of at least 50 cells for each experimental group. In detail, in order to examine the whole sectioned cell pellet, the grid was scanned in equally spaced parallel sweeps starting from a grid square corner.

Mitochondria were identified based on the presence of an inner and an outer membrane, an internal matrix, and a system of crests. Although the morphology of mitochondria may vary also in physiological conditions, altered mitochondria are defined according to ultrastructural criteria, which were validated in previous studies [12,19]. The number of mitochondrial alterations was used to provide a novel scoring system (modified from Flameng and coll. [23]), which was extensively reported in the results section. For the purpose of clarity, it may be summarized here as follows: score 1, mitochondria with spread matrix dilution and membrane alterations concerning both crests and inner/outer membranes; score 2, mitochondria with spots of matrix dilution with some membrane alterations in the form of broken crests; score 3, mitochondria with intact membranes but owing spots of matrix dilution; score 4, intact mitochondria.

### 2.7. Post-Embedding Immuno-Electron Microscopy

Ultra-thin sections were collected on nickel grids, they were de-osmicated in aqueous saturated solution of sodium metaperiodate (NaIO_4_) for 15 min, washed three times in ice cold filtered PBS, (pH 7.4) for 10 min, and then treated with ice-cold PBS containing 10% goat serum and 0.2% saponin to block non-specific antigens for 20 min at 21 °C.

Primary antibodies were incubated in ice-cold PBS containing 1% goat serum and 0.2% saponin in a humidified chamber overnight, at 4 °C. The following primary antibodies were used: anti-LC3 (1:50, Abcam); anti-Cat D (1:10, Sigma-Aldrich, Saint Louis, MO, USA); anti-Prion Protein (PrP, 1:10, Chemicon, Temecula CA, USA); anti-PARK (1:20, Millipore); anti-Pink1 (1:20, Abcam). Solutions containing two primary antibodies were used in order to detect co-localization of LC3 and Cat D proteins.

Pre-treatment of the sample with proteinase K (PK, Sigma-Aldrich, Saint Louis, MO, USA, 50 mg/mL) allows to digest all properly folded, non-aggregated proteins, including native PrPc. Based on our previous study [24], PK tratment does not alter the ultrastructure of the cells. In this way, primary antibody against PrPc, when staining PK-resistant protein, documents the presence of scrapie-like prion protein (PrPsc-like). The treatment of the sample with PK was carried out with the specific purpose to carry out a quantitative ultrastructural stoichiometry in situ, which allowed optimizing detection and localization of either PrPc or PrPsc-like proteins within GBM cells. Therefore, sample grids were combined with drops of PK solution (50 mg/mL) in 0.1M PBS for 1 h at 37 °C. Proteinase K digests.

All primary antibodies were revealed through a solution containing gold-conjugated secondary antibodies (gold particle diameter, 10 nm or 20 nm, BB International, Cardiff UK) diluted 1:10, in PBS containing 1% goat serum and 0.2% saponin for 1 h, at room temperature. After rinsing in PBS, grids were incubated in 1% glutaraldehyde for 3 min, washed in distilled water, and further stained with uranyl acetate and lead citrate. Ultra-thin sections were finally observed at Jeol JEM SX100 TEM (Jeol). Control sections were incubated with secondary antibodies only.

### 2.8. Western Blotting

To analyze autophagy flux U87MG and A172 cells were treated with 10 nM rapamycin or vehicle (control) for 24 h immediately followed by cell culture washed out. Cells were lysed immediately after treatment or following 4, 7, or 14 days of rapamycin withdrawal. Bafilomycin A1 (100 nM; Sigma-Aldrich, Saint Louis, MO, USA) was added to the GBM cells alone or in combination with rapamycin during the last 3h before cell lysis. At the end of each time interval, cells were lysed for western blotting and electrophoretically resolved as previously reported [25]. These proteins were electro-transferred onto PVDF membranes (BioRad Laboratories, Hercules, CA, USA) by a semi-dry system (BioRad Laboratories, Hercules, CA, USA). Membranes were blocked with 3% non-fat milk in PBS containing 0.1% Tween-20 (TBST) and then incubated (overnight at 4 °C) with the following antibodies: anti-LC3-I and LC3-II (MBL International, Woburn, MA, USA), anti-p62/SQSTM1 (Sigma-Aldrich, Saint Louis, MO, USA). After extensive washing with TBST, blots were incubated with a 1:3000 dilution of HRP-conjugated secondary antibody (Amersham Biosciences, Amersham, UK) for 1 h, at room temperature. Immunostained bands were detected with naked eye, and they were measured by using the classic non-quantitative, non-necessarily linear quantification through chemiluminescence (GE Healthcare Biosciences, Little Chalfont, Buckinghamshire, UK). Membranes were probed with the housekeeping mouse anti-β-actin (1:25,000, Sigma-Aldrich, Saint Louis, MO, USA). Densitometric analysis of p62/β-actin and LC3-II/LC3-I ratio was performed with ImageJ software (NIH, Bethesda, MD, USA, Version 1.8.0_172).

To analyze mTOR inhibition under the effect of rapamycin the downstream PS6RP was evaluated. Autophagy activation was evaluated by measuring the levels of the early autophagy stimulating protein VPS34. Finally, Fis1 and DRP1 were measured as markers of mitochondrial fission and mitophagy. U87MG cell pellet was placed in an Eppendorf tube containing 20 μL of ice-cold lysis buffer with phosphatase and protease inhibitors to be homogenized. An aliquot of the homogenate was used for Bradford protein assay. Proteins (20 μg) were separated on SDS-polyacrylamide gels (Mini Protean TGX precast gel 4–20% gradient, BioRad Laboratories, Hercules, CA, USA) on Trans-blot Turbo Transfer System Pack (for mixed molecular weight; 1.3 A-25 V-10 min). Membranes were blocked for 2 h in Tween-20 Tris-buffered saline (TTBS) (100 mM Tris-HCl, 0.9% NaCl, 1% Tween 20, pH 7.4) containing 5% non-fat dry milk (BioRad Laboratories, Hercules, CA, USA). We used the following primary antibodies: (i) anti-PS6RP (1:1000, Cell Signaling); (ii) anti-VPS34 (1:1000, Thermo-Fisher Scientific, Waltham, MA, USA); (iii) anti-Fis1 (1:1000, GeneTex); (iv) anti-DRP1 (1:2000, Abcam). Rabbit anti-β-actin (1:50,000; Sigma-Aldrich, Saint Louis, MO, USA) was used as an internal standard for semi-quantitative protein measurement.

Membranes were incubated overnight at 4 °C with primary antibodies diluted in TTBS containing 2.5% non-fat dry milk, and then they were washed in TTBS and incubated for 1 h with peroxidase-labeled secondary antibodies (anti-rabbit/anti-mouse, 1:3000; Calbiochem, Milan, Italy). Bands were visualized with enhanced chemiluminescence reagents (GE Healthcare, Milan, Italy). Image analysis was carried out by ChemiDoc System (BioRad Laboratories, Hercules, CA, USA). Optical density was normalized for relative β-actin using Image J software (NIH, Bethesda, MD, USA, Version 1.8.0_172).

### 2.9. Cell Cycle Analysis

U87MG cells in logarithmic growth were treated with 10 nM rapamycin or vehicle. After 24 h a part of the sample was immediately prepared for flow cytometry, while the rest was maintained in culture for 4 days, 7 days, and 14 days without any further treatment. At the end of each incubation time, samples were fixed with 70% ethanol, stained with 20 μg/mL of propidium iodide and 100 μg/mL of RNase A, and then incubated at 37 °C for 30 min. Flow cytometric analysis was performed with appropriate gating on a FACScan (Becton Dickinson, Milan, Italy).

### 2.10. Statistical Analyses

The optical density of MTR-G histo-fluorescence and each protein immuno-fluorescence (PS6RP, LC3, Cat D, Pink1, PARK, BNIP3, Fis1 and DRP1) was given as mean percentage ± SEM per cell from N = 100 cells/group (assuming controls as 100%).

The merging of immuno-fluorescent puncta was counted and expressed as the mean ± SEM of puncta per cell in each experimental group (each group being representative of a specific dose and timing of rapamycin/vehicle saline) from N = 100 cells.

The optical density of immuno-blotting was given as mean ± SEM from 2 ≤ N ≤ 6 samples per experimental group.

Flow cytometry values were expressed as mean percentage ± SEM of cells in each cell cycle phase (G_1_, S, or G_2_+M).

For ultrastructural morphometry, values were expressed as following: (i) total number of mitochondria per cell; (ii) percentage of altered mitochondria per cell; (iii) number of Pink1- or PARK-positive mitochondria per cell; (iv) number of LC3- and/or Cat D-positive vacuoles per cell, (v) number of Pink1 or PARK immuno-gold particles per cell; (vi) number of Pink1 or PARK immuno-gold particles within mitochondria; (vii) the ratio between the number of Pink1 or PARK immuno-gold particles within mitochondria and the number of Pink1 or PARK immuno-gold particles within cytosol; (viii) number of PrPc or PrPsc-like immuno-gold particles per cell.

Values are given as the mean number or the mean percentage ± SEM per cell from 50 cells per group in all counts.

Mitochondrial score was calculated by averaging mitochondrial scores (as established from the first block of Results, in the Results section) from at least 150 mitochondria from each experimental group. Values are given as the mean ± SEM.

Data are compared using ANOVA with the Scheffe’s post-hoc test or Bonferroni’s corrected *t* test (for cell cycle data). Differences between the various groups are considered to be significant when the null hypothesis H_0_ is less than 5%.

## 3. Results

### 3.1. Rapamycin Induces a Marked, Long-Lasting mTOR Inhibition

**Figure 1:**
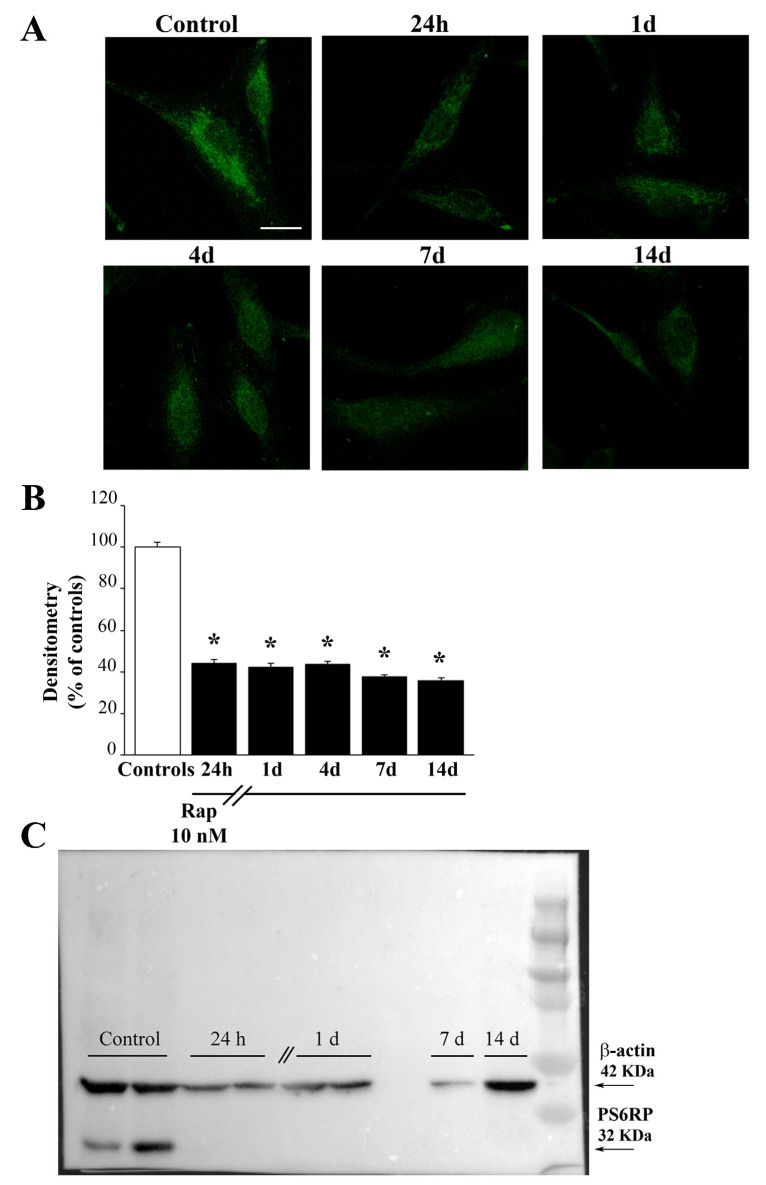
Rapamycin produces a long-lasting inhibition of mTOR activity. (**A**) Representative pictures showing the immunofluorescence of the protein PS6RP, which is a downstream enzymatic product of mTOR. Rapamycin produces a marked and long-lasting decrease of the PS6RP immunofluorescence. Densitometry of the PS6RP immunofluorescence (**B**) and the representative PS6RP immunoblotting (**C**) indicate that rapamycin induces a massive mTOR inhibition, which lasts at least 14 days following rapamycin withdrawal. Data in (**B**) are given as the mean percentage ± SEM of optical density measured in 100 cells per group (assuming controls as 100%). * *p* < 0.05 compared with controls. Scale bar = 12 μm.

As expected from cell lines featuring mTOR overexpression, detectable amount of the enzymatic product PS6RP is measured in baseline GBM cells. This amount is persistently suppressed by a short-lasting rapamycin administration. In fact, mTOR suppression was measured at least for 14 days following rapamycin withdrawal (Figure 1).

As shown in Figure 1 and Figure S1, when rapamycin is administered in these experimental conditions it occludes the enzymatic activity of mTOR in both U87MG and A172 cells, respectively. In fact, following 24 h rapamycin exposure, and after washing rapamycin-treated cells, following rapamycin withdrawal from 24 h up to 14 days, the presence of the downstream product of mTOR, PS6RP is persistently suppressed, and it is found in a negligible amount compared with GBM cells administered vehicle. The persistent suppression of the mTOR activity following rapamycin withdrawal is even more pronounced considering P70S6K, the direct mTOR substrate, as reported in Supplementary Figures S2 and S3, which show the P70S6K immunofluorescence in U87MG and A172 cells, respectively. Such a long-lasting effect indicates that inhibition in the activity of mTOR in rapamycin-treated cells is consistent along prolonged time intervals. These findings are key to interpret the effects induced by rapamycin to various proteins and mitochondria from GBM cells, as reported in the following paragraphs.

### 3.2. Autophagy Flux

**Figure 2:**
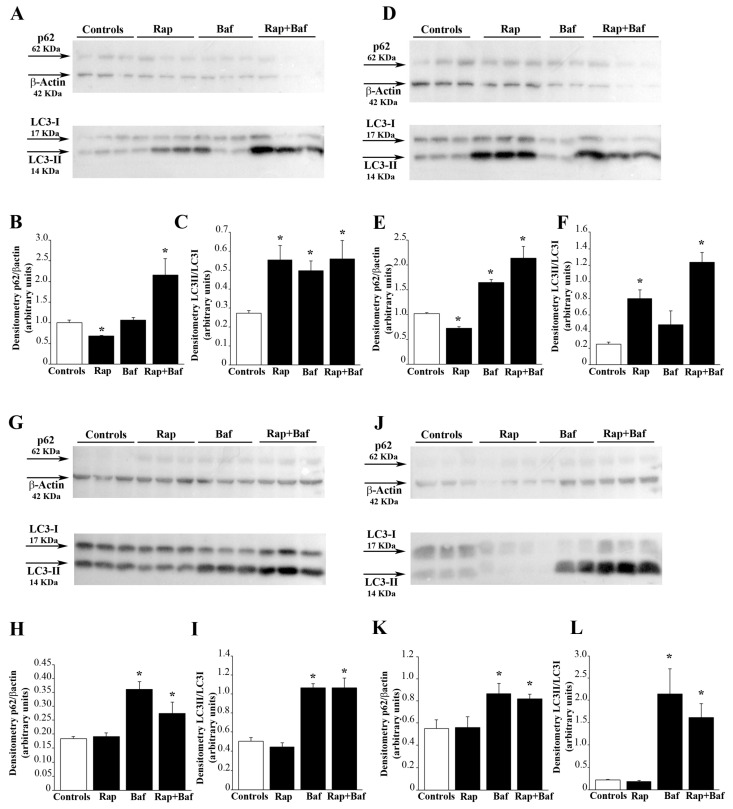
Rapamycin treatment increases the autophagy flux. Western blot analysis of p62 and LC3-II in U87MG cells treated with 10 nM rapamycin for (**A**) 24 h and (**D**) 4 d, (**G**) 7 d and (**J**) 14 d after its removal. In some samples bafilomycin A1 (100 nM) was added during the last 3 h in both untreated and rapamycin-treated cultures. Densitometry analysis of the level of p62 compared with the housekeeping β-actin and LC3-II compared with LC3-I is also reported in the graphs. In detail, densitometry of p62/β-actin in cells treated with rapamycin for (**B**) 24 h and (**E**) 4 d, (**H**) 7 d and (**K**) 14 d after its removal is reported. Similarly, densitometry of LC3-II/LC3-I in cells treated with rapamycin for (**C**) 24 h and (**F**) 4 d, (**I**) 7 d and (**L**) 14 d after its removal is shown. Values are given as the mean ± SEM from three samples per experimental group. * *p* < 0.05 compared with controls.

As expected, due to inhibition of mTOR, rapamycin rescues the autophagy flux which is known to be suppressed in baseline GBM cells. In fact, as shown in the blotting of Figure 2A, p62 levels are reduced following rapamycin. The measurement of this effect is reported in the graph of Figure 2B showing that, p62 levels are roughly 30% of untreated control. Besides, when bafilomycin is added during the last 3 h of rapamycin treatment, a significant reduction in p62 levels and a concomitant increase in LC3-II occurs compared with bafilomycin alone (Figure 2B,C). Taken together, these results indicate that rapamycin increases the autophagy flux. It is remarkable that, following 24 h of rapamycin administration, after rapamycin being washed out, the stimulation of the autophagy flux persists for several days of withdrawal. In detail, at four days following rapamycin withdrawal, the levels of p62 are still significantly lower than controls (Figure 2E). When bafilomycin is added at four days following rapamycin withdrawal results are similar to that observed at 24 h (compare Figure 2E,F with Figure 2B,C). At seven days following rapamycin withdrawal, the level of p62 remains below the level of controls (Figure 2H), along with a significant decrease in LC3-II levels (Figure 2I). At 14 days following rapamycin withdrawal, the decrease in p62 persists (Figure 2K) while the level of LC3-II is rescued (Figure 2L). Similar findings are obtained when the autophagy flux was measured in A172 cells following 24 h of rapamycin exposure (Supplementary Figure S4), and 4 d (Supplementary Figure S5), 7 d (Supplementary Figure S6), and 14 d (Supplementary Figure S7) after rapamycin withdrawal.

### 3.3. Rapamycin Promotes the Merging of LC3 with Cathepsin D

**Figure 3:**
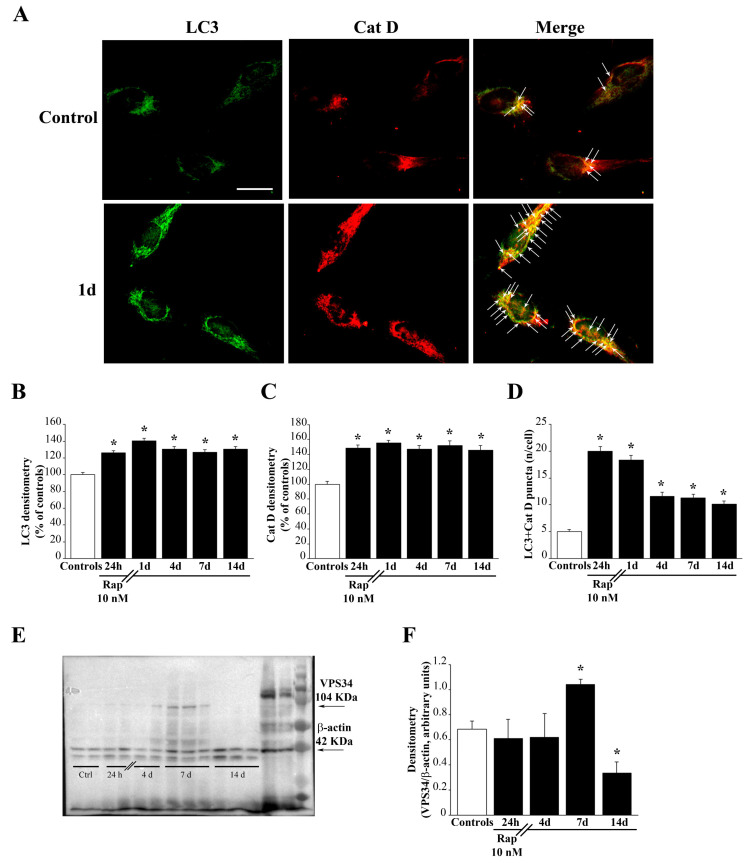
Rapamycin induces autophagy. (**A**) Representative light microscopy of the autophagy markers LC3 and Cat D, showing that rapamycin induces immuno-fluorescent puncta for LC3 + Cat D. Arrows point to merging (yellow) puncta. Graphs report (**B**) LC3 fluorescent density (**C**) Cat D fluorescent density, and (**D**) LC3 + Cat D merging puncta. Counts report means ± SEM from three independent experiments. (**E**) Representative western blotting of the autophagy protein VPS34 at different time intervals following rapamycin. Graph in (**F**) reports that, following rapamycin, an increased expression of VPS34 was measured at 7 days of rapamycin withdrawal. Values are given as the mean ± SEM of optical density measured from an average of at least four samples per group. * *p* < 0.05 compared with controls. Scale bar = 20 μm.

The stimulatory effects of rapamycin on the autophagy flux are validated by assessing the merging between autophagosomes and lysosomes markers. This is shown by immuno-fluorescent puncta in Figure 3 and Supplementary Figure S8, where the green fluorescence of LC3 merge with the red fluorescence of Cat D, way in excess in rapamycin-treated cells compared with controls. This is shown in representative pictures of Figure 3A for U87MG cells and Supplementary Figure S8A for A172 cells. Again, such an effect lasts at least for two weeks following rapamycin withdrawal, as shown in the graphs of Figure 3B,C reporting fluorescence densitometry for each antigen, and as counted in the graph of Figure 3D where the number of merging puncta is reported. Similar results are obtained in A172 cells (Supplementary Figure S8B,C, respectively). This is confirmed in Supplementary Figures S9 and S10, which refer to U87MG and A172 cell cultures, respectively. These graphs report the actual counts of stained autophagosomes and lysosomes which are counted at ultrastructural morphometry along with TEM counts of their merging in autophagolysosomes. In fact, these graphs report that rapamycin increases the number of LC3 positive vacuoles (autophagosomes) identified at ultrastructural morphometry along with the number of Cat D positive vacuoles (lysosomes). Most remarkably, rapamycin administration peristently increases the merging of these compartments counted as LC3 + Cat D positive vacuoles (autophagolysosomes). This provides gold-standard, direct evidence for a rapamycin-induced acceleration of the autophagy flux.

### 3.4. Rapamycin Induces the Expression of VPS34

To implement data regarding rapamycin-induced stimulation of the autophagy flux, we checked whether rapamycin was effective in increasing early autophagy markers. Interestingly, although rapamycin was not effective at early time intervals in increasing the early autophagy protein VPS34, this effect was evident at delayed time intervals, starting at seven days to decline at 14 days following rapamycin exposure. At seven days, rapamycin produces a remarkable increase in the early autophagy stimulating protein VPS34. This is shown in Figure 3E,F. VPS34 is one of the earliest autophagy-related proteins, which contributes to start the autophagy pathway. It is remarkable that such a stimulatory effect of rapamycin on VPS34 is delayed and transient compared with the autophagy flux (Figure 3E,F).

### 3.5. Rapamycin Shift Cell Cycle towards G1

**Figure 4:**
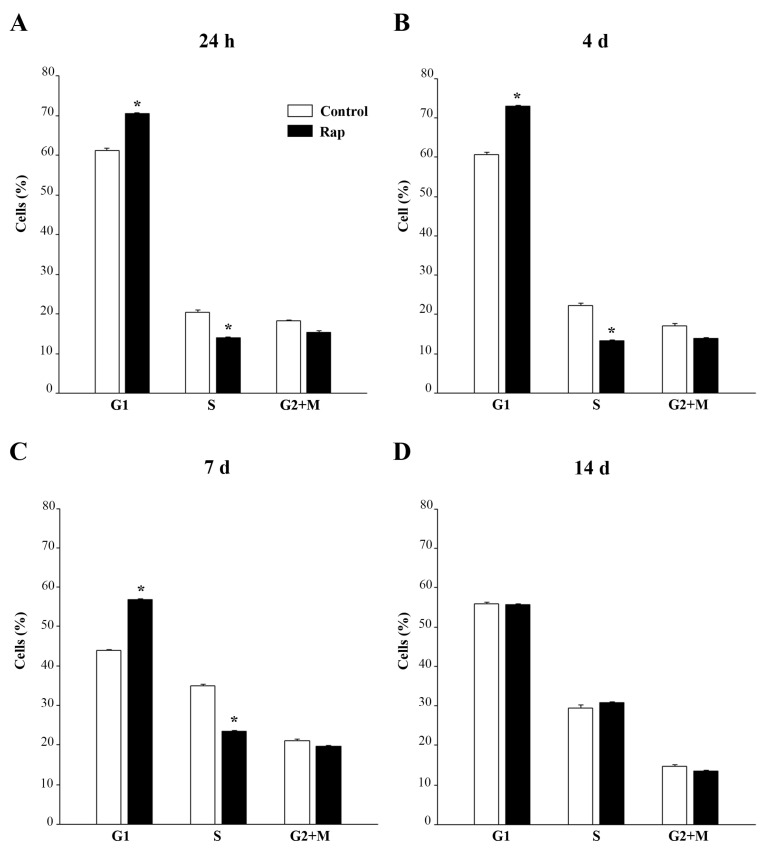
Rapamycin induces a long lasting G1 arrest. U87MG cells were treated with 10 nM rapamycin and the cell cycle was analysed after 24 h (**A**) and at 4 days, 7 days, 14 days after rapamycin removal (**B**–**D**, respectively). Values are given as the mean ± SEM from three independent experiments. * *p* < 0.05 compared with controls.

The cell cycle analysis by flow cytometry shows that 24 h rapamycin (10 nM) increases GBM cells in the G1 phase compared with controls with a concomitant decline in the percentage of cells in the S phase (Figure 4A). It is remarkable that, as for data measuring the autophagy flux, rapamycin-induced alteration of the cell cycle persists at long time intervals after drug washout/withdrawal. In fact, similar results were obtained at 24 h, four days, and seven days after rapamycin withdrawal (Figure 4A–C, respectively).

### 3.6. Rapamycin Decreases Cytoplasmic Prpc and PrPsc-like Proteins

**Figure 5:**
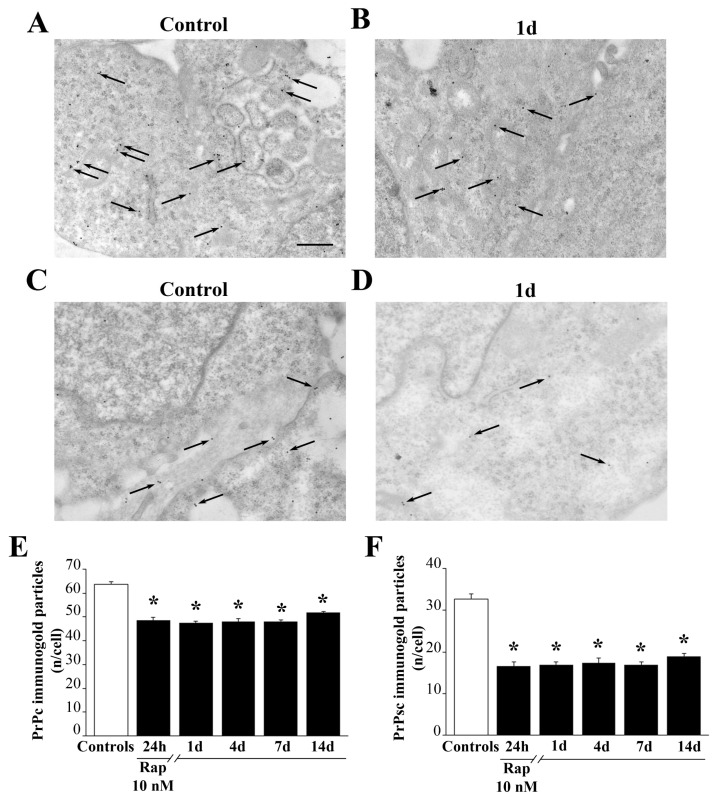
Rapamycin decreases cytoplasmic PrPc and PrPsc-like proteins. Representative TEM micrographs showing a PrPc- or PrPsc-like stained control cell (**A**,**C**) respectively and a cell following 1d of rapamycin withdrawal (**B**,**D**) respectively. Arrows point to PrPc/PrPsc-like immuno-gold particles within cell cytosol. Quantitative ultrastructural morphometry for PrPc (**E**) and PrPsc-like (**F**) immuno-gold particles within cytosol is reported in the graphs. Counts represent the mean ± SEM from N = 50 cells per group. * *p* < 0.05 compared with control. Scale bar = 230 nm.

The occurrence of PrPc was elevated in baseline conditions in GBM cells. When ultrastructural stoichiometry was carried out, the authetic number of PrPc and PK resistant PrPsc-like particles were detected both in control conditions and at various time intervals following 24 h exposure to rapamycin. As shown in representative Figure 5, PrPc (Figure 5A) and PrPsc-like (Figure 5C) were more abundant in control GBM cells and they decrease following rapamycin (Figure 5B for PrPc and Figure 5D for PrPsc-like). It is rermarkable that the amout of both prion proteins were more abundant at the level of the plasma membrane compared with the whole cytosol. As shown in the graphs of Figure 5E,F, the suppression of prion protein levels was significantly induced by rapamycin up to 14 days of withdrawal. The rapamycin-induced suppression was much more relevant concerning the PK resistant PrPsc-like isoform (graph of Figure 5F) compared with the PrPc isoform (graph of Figure 5E). Similarly, PrPc and PrPsc-like decrease in A172 cells treated with rapamycin compared with control cells (Supplementary Figure S11) and such a suppression is long lasting and more marked for the PK resistant isoform (Supplementary Figure S12).

### 3.7. Rapamycin Persistently Increases Mitochondrial Number and Improves Mitochondrial Structure

**Figure 6:**
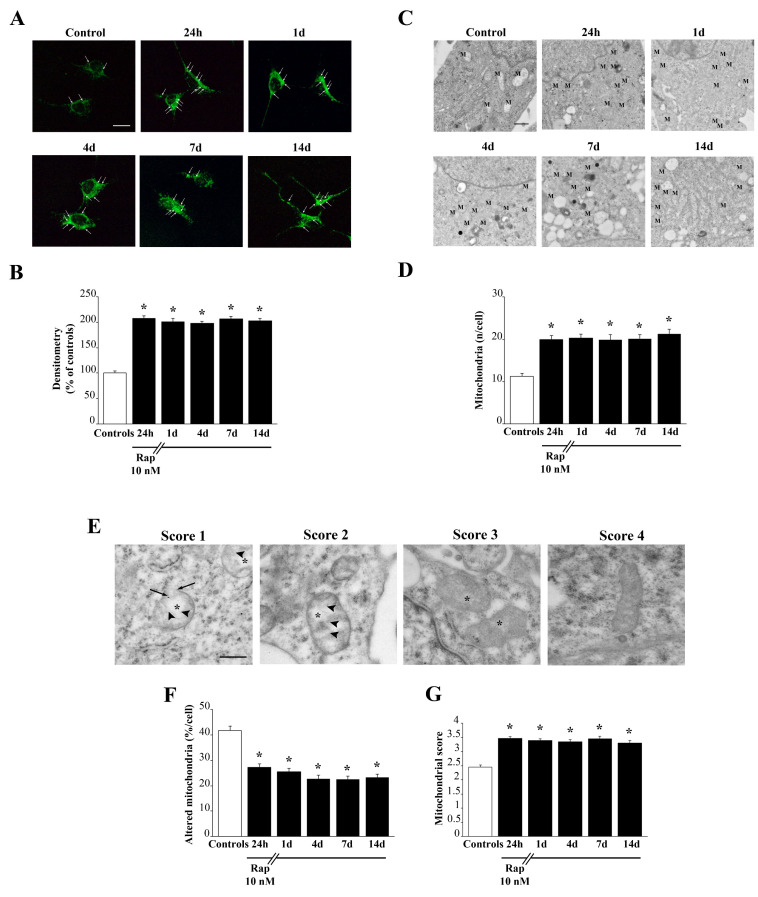
Rapamycin persistently increases the number of mitochondria and produces a long-lasting rescue of altered mitochondria. (**A**) Representative pictures show total mitochondria stained with MTR-G from controls and following rapamycin treatment (10 nM) for 24 h and after rapamycin withdrawal (up to 14 d), Arrows indicate intensely fluorescent signals. (**B**) Graph reports the two-fold steady increase in mitochondrial MTR-G fluorescence induced by rapamycin, which persists unmodified for 14 days. Values are given as the mean ± SEM from N = 100 cells per group. (**C**) Representative pictures at TEM show the increase in mitochondria (M) counted directly as specific organelles. (**D**) Graph reports the count of mitochondria per cell at TEM which overlaps the two-fold increase measured at MTR-G fluorescence, which similarly persists at 14 days. Values are given as the mean±S.E.M from N = 50 cells per group. (**E**) Representative pictures depicting mitochondrial morphology evaluated based on the modified scale from Flameng et al. 1980 [23]. A score of 1 indicates that mitochondria possess broken crests (arrowheads) with ruptured mitochondrial membranes (arrows), and a spread matrix dilution (asterisk); a score of 2 indicates a mitochondrion that possess broken crests with spots of diluted matrix but membranes intact; a score of 3 indicates that mitochondria possess only spots of diluted matrix but intact crists and intact inner/outer membranes; the score of 4 indicates an intact mitochondrion. Graph (**F**) reports the percentage of altered mitochondria (including all kinds of alterations). Graph (**G**) reports the average of mitochondrial score. Values are given as (**F**) the percentage ± SEM from N = 50 cells per group or (**G**) the mean mitochondrial morphological score ± SEM calculated by averaging mitochondrial scores from at least 150 mitochondria from each experimental group. * *p* < 0.05 compared with controls. Scale bars: (**A**) = 18 μm; (**C**) = 260 nm; (**E**) = 230 nm.

As shown in Figure 6, rapamycin induces a long-lasting increase in mitochondria. This is evident both in representative pictures of MTR-G histofluorescence (Figure 6A) and direct imaging of mitochondria at TEM (Figure 6C). The amount of such an increase is similar at light microscopy and electron microscopy. In fact, both methods report an increase of mitochondrial imaging of roughly two-fold of controls (compare graphs of Figure 6B,D). MTR-G fluorescence and direct visualization and quantification of total mitochondria at TEM carried out in A172 cells following various times of rapamycin withdrawal provided very similar results (Supplementary Figures S13 and S14, respectively). While light microscopy with MTR-G does not provide any information concerning mitochondrial integrity, this is positively assessed at TEM as reported in Figure 6C,E. In detail, representative micrographs of Figure 6E show that rapamycin decreases altered mitochondria by improving mitochondrial structure concerning matrix dilution, integrity of crests, and mitochondria membranes. This is counted in the graph of Figure 6F, which reports a significant decrease in the number of altered mitochondria per cell (where altered mitochondria are considered independently from the kind of specific alteration). The number of altered mitochondria is reduced by rapamycin to roughly 50% of controls. Remarkably, such an effect persists at 14 days of rapamycin withdrawal. This overalaps with data obtained by scoring mitochondrial integrity, where the highest values correspond to intact mitochondria as reported in the graph of Figure 6G. The graph reports a long-lasting, rapamycin-dependent increase in the mitochondrial integrity score. Such an integrity score allows to detail the specifc mitochondrial alterations which may sum up to provide a severity score for mitochondrial alterations. In detail, such a score attributes four points to intact mitochondria; three characterizes mitochondria with intact membranes but owing spots of matrix dilution; two is the score given to mitochondria owing spots of matrix dilution with some membrane alterations in the form of broken crests. The score of one is mostly severe and corresponds to mitochondria where matrix dilution is spread and membrane alterations concern both crests and inner/outer membranes. In this way, the graph in Figure 6G is more specific since it provides both the kind and the severity of mitochondrial alterations. Both graphs in Figure 6F,G indicate that rapamycin persistently improves mitochondrial integrity. Thus, apart from increasing two-fold the number of mitochondria, rapamycin specifically increases the number of healthy mitochondria. Again, mitochondrial alterations and mitochondrial integrity score measured in A172 cells (Supplementary Figure S15) are in line with those measured in U87MG cells.

### 3.8. Rapamycin Increases the Merging of LC3 with MTR-G

**Figure 7:**
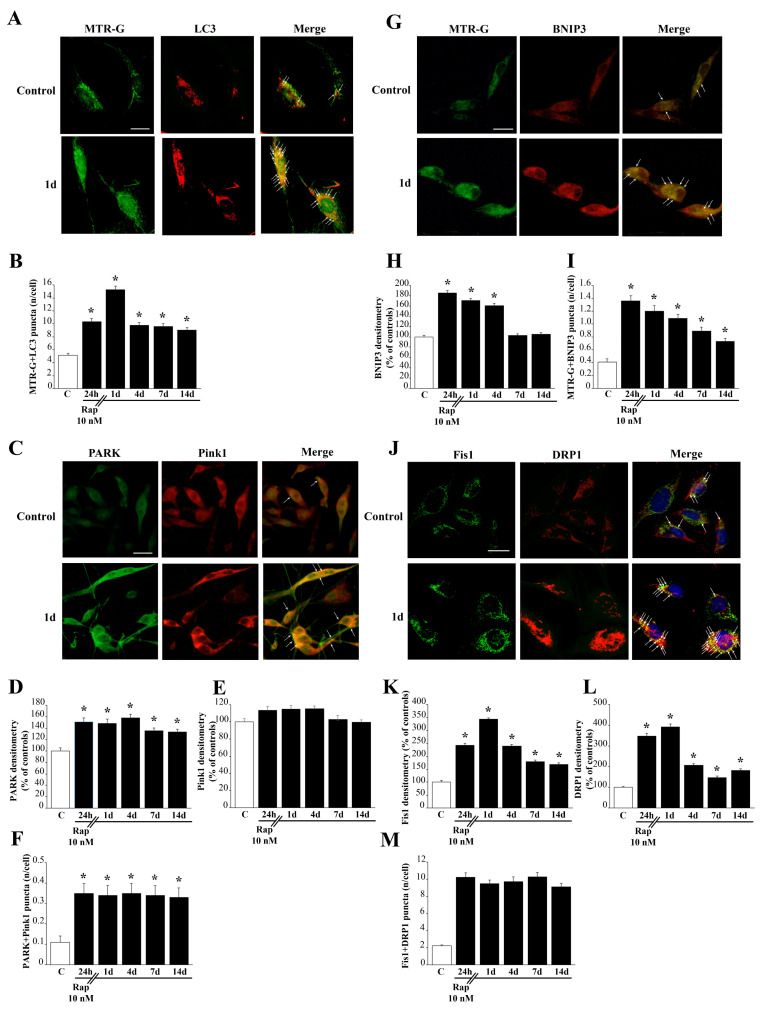
Rapamycin increases autophagy-related mitochondrial dynamics. (**A**) Representative pictures show that rapamycin increases histofluorescence of MTR-G and immuno-fluorescence for LC3. Arrows point to merging (yellow) puncta. (**B**) Graph reports the rapamycin-induced long-lasting increase in MTR-G+LC3 merging puncta. (**C**) Representative pictures show mitophagy markers PARK and Pink1 from control and rapamycin-treated cells (10 nM at 1 day). Arrows point to merging (yellow) puncta. (**D**) The graph of PARK immunofluorescence indicates a long-lasting significant increase following rapamycin. (**E**) The graph shows no significant effect of rapamycin on Pink1 immunofluorescence at any time interval. (**F**) The graph indicates a significant increase in the number of puncta showing the merging of PARK and Pink1, which exceeds at large the increase induced in PARK immunofluorescence. (**G**) Representative pictures show that rapamycin increases histofluorescence of MTR-G and immunofluorescence for BNIP3 compared with control. Arrows point to merging (yellow) puncta. (**H**) Graph reports the rapamycin-induced long-lasting increase in BNIP3 immunofluorescence. (**I**) Graph reports the rapamycin-induced long-lasting increase in MTR-G+BNIP3 merging puncta. (**J**) Representative pictures of the fission markers Fis1 and DRP1. Arrows point to merging (yellow) puncta. Graphs (**K**) and (**L**) report Fis1 and DRP1 immunofluorescent intensity, respectively. Graph (**M**) reports Fis1 + DRP1 merging puncta. Values are given as the mean±S.E.M from N = 100 cells per group. C=Controls * *p* < 0.05 compared with controls. Scale bars: (**A**) = 13 μm; (**C**) = 20 μm; (**G**) = 18 μm; (**J**) = 13 μm.

As shown in representative Figure 7A and Figure S16, rapamycin increaseas both MTR-G histo-fluorescence and LC3 immuno-fluorescence in U87MG and A172 cells, respectively. Most importantly, following 1 day of rapamycin withdrawal a remarkable merging of MTR-G with LC3 fluorescence is evident. This is measured in the graph of Figure 7B, which reports a robust increase of merging puncta per cell, where MTR-G and LC3 are concomitanly present. This effect persists significantly for two weeks following rapamycin withdrawal.

### 3.9. Rapamycin Increases the Merging of Parkin (PARK) and Pink1

As shown in representative pictures of Figure 7C, rapamycin increases the PARK immuno-fluoresence, while leaving intact Pink1 immunofluorescence. This is counted in the graph of Figure 7D, which reports a long-lasting increase in PARK immuno-fluorescence which persists unmodified at least for 14 days of rapamycin withdrawal. In contrast, as reported in the graph of Figure 7E, Pink1 immunofluorescence is not modified compared with controls by rapamycin admnistration at any time interval. When double immunoflurescence is calculated, as reported in the graph of Figure 7F, a significant increase, which persists at least 14 days is measured for the merging of PARK and Pink1 immunofluorescence following rapamycin 10 nM. Such an increase in merging fluorescence exceeds at large the increase induced by rapamycin in single PARK immunofluorescence. When considering that Pink1 immunofluorescence is not modified by rapamycin, the more marked increase in double immunofluorescence compared with single PARK immunofluorescence indicates that the increase in PARK following rapamycin occurs with a selective placement with a site-specificity depending on where baseline Pink1 immunofluorescence occurs. This specific placement is expected to correspond to rapamycin-responsive mitochondria as suggested by previous studies. We confirmed this here using TEM (Supplementary Figures S17 and S18). These data were replicated in A172 cells (Supplementary Figure S19, for PARK and Pink1 double immunofluorescence and Supplementary Figures S20 and S21, for PARK and Pink1 immunogold at TEM, respectively).

### 3.10. Rapamycin Increases the Merging of MTR-G with BNIP3

As shown in representative pictures of Figure 7G, rapamycin increases the immuno-fluorescence of BNIP3 and MTR-G fluorescence. Immunofluorescence for BNIP3 is measured in the graph of Figure 7H, which reports a long-lasting (four days) increase in immunofluorescence during rapamycin withdrawal. When double fluorescence was calculated, as reported in the graph of Figure 7I, MTR-G+BNIP3 fluorescent puncta are increased by rapamycin for at least 14 days. This is likely to depend on the long-lasting increase in MTR-G (as measured in the graph of Figure 6B) joined with the persistence of elevated BNIP3 specifically at mitochondrial level. This is expected based on the selective increase of such a mitophagy-related protein focally within mitochondria. Similar results are obtained for MTR-G and BNIP3 fluorescence carried out in A172 cells (Supplementary Figure S22).

### 3.11. Rapamycin Increases Immunofluorescence for Fis1 with DRP1

Rapamycin 10 nM exposure increases the markers of mitochondrial fission Fis1 and DRP1 as shown in representative pictures of Figure 7J. In detail, as shown in the graph of Figure 7K, Fis1 increases markedly at short-time intervals, and it remains elevated at least 14 days following rapamycin withdrawal. Similarly, as shown in the graph of Figure 7L, DRP1 markedly increases at short time-intervals following rapamycin exposure, although it remains significantly elevated at delayed time intevals (Figure 7L). This trend is confirmed by Western blotting reported in Supplementary Figure S23 for Fis1 and Supplementary Figure S24 for DRP1. In fact, Fis1 protein is increased for 14 days of rapamycin withdrawal as well as DRP1. However, the increase in DRP1 occurs more markedly at early time intervals. When counting merging puncta, the increase of puncta is quite steady up to 14 days, as reported in the graph of Figure 7M. This indicates that, despite the dramatic increase in each fission marker occurring only at early time intervals, their co-localization persists unmodified for at least 14 days. This suggests that the amount of both antigens, which are slightly elevated at prolonged time intervals, is responsible for merging and it may reflect a specific mitochodrial cell compartment. Fis1 + DRP1 immunofluorescence and counts of Fis1 + DRP1-positive puncta provided similar results in A172 cells (Supplementary Figure S25).

## 4. Discussion

The present study, which is validate in different cell lines, indicates that, mTOR inhibition, which is evidenced by suppressed PS6RP and P70S6K expression, induced by rapamycin in the therapeutic range (10 nM, [20]) produces a sudden and concomitant activation of the autophagy flux as measured by the classic assay with bafilomycin, the merging of LC3 with Cat D immunofluorescence, and the merging of autophagosomes with lysosomes by gold standard TEM. Most of these effects persist unmodified at least 14 days following rapamycin withdrawal and they are concomitant with a shift of a significant amount of GBM cells from S towards G1 phase, as measured by flow cytometry. These effects are long-lasting since they extend long beyond rapamycin withdrawal, for at least 14 days. Remarkably, this is concomitant with a marked suppression in the amount of the cellular prion protein, which is detected at molecular level considering the stoichiometry of both native PrPc and the protease resistant isotype PrPsc-like. These effects are tightened with an increase in the total number of mitochondria detected using both light (MTR-G fluorescence) and electron microscopy (direct count of the organelles). The increase in mitochondria occurs although the number of altered mitochondria and the number of mitochondrial alterations are steadily suppressed, at least for 14 days. This indicates a robust improvement in mitochondrial integrity. The autophagy-related increase in mitochondria is confirmed by the merging of LC3 with MTR-G, which increases following rapamycin administration. Similarly, the significance of increased LC3 is validated concerning the autophagy flux within different GBM cell lines, by using bafilomycin assay and the gold standard assessment of autophagy status at TEM. In fact, TEM also indicates that rapamycin stimulates the merging of autophagosomes with lysosomes, which is backed up by increased merging of LC3 with Cat D immuno-fluorescence. A marked effect of rapamycin in situ on mitochondrial proteins is confirmed by the merging of fluorescence for the mitophagy protein BNIP3 with MTR-G. This is further witnessed by AN increased amount and augmented merging of the mitophagy-related proteins Pink1 and PARK which is shown here at immunofluorescence and electron microscopy. This latter evidence confirms in GBM cells the effects of the prion proteins on suppressing Pink1/PARK interactions which were recently evidence in prion disorders [17]. This is concomitant with an increase in the mitochondrial fission/mitophagy-related proteins Fis1 and DRP1, which persists at least for 14 days following rapamycin withdrawal. This is in line with the well-established concept that when promoting global autophagy, mitochondrial fission and mitophagy concomitantly increase [26]. These data confirm a long-lasting effect following rapamycin withdrawal [19] and indicate a powerful and persistent activation of autophagy and mitophagy under the effects of rapamycin. In detail, the long-lasting effects of rapamycin on the autophagy flux in GBM cells were not described previously. The stimulation of the autophagy flux, which is estimated by counting the amount of LC3-II and p62 with or without bafilomycin, persists during rapamycin withdrawal. This is concomitant with the merging of LC3 with Cat D immunofluorescence and the fusion of autophagosomes with lysosomes. The increase in proteins, which promote mitochondrial biogenesis explains the increase in healthy mitochondria reported here, which confirms previous recent data [12]. In this recent report, evidence was provided that rapamycin increases the expression of those genes promoting mitochondrial biogenesis at 14 days following rapamycin withdrawal. In line with this, the present study indicates that rapamycin promotes a persistent clearance of PrPc, which takes place at least for 14 days following rapamycin withdrawal. Again, PrPc removal is consistent with enhanced autophagy flux, which efficiently clears such an aggregate prone protein mostly concerning its PK-resistant isoform.

Long-lasting improved mitochondrial turnover joined with persistent suppression of prion protein levels in GBM is likely to explain the shift of cell cycle from S towards G1 phase. Accordingly, a shift in the balance between mitochondrial fission and fusion can modulate the progression of the cell cycle [27] and different phases of the cell cycle are associated with changes in mitochondrial status [28]. In detail, the S to G1 transition is concomitant to increased mitochondrial fission [28], while the opposite change from G1 to S phase is accompanied by increased mitochondrial fusion [29].

In line with concomitant autophagy activation, the increase in DRP1 (fission protein) produces increased mitochondrial turnover with fragmentation of altered mitochondria [30] and mitochondria biogenesis, which is evidenced by the presence of elongated mitochondria owing to densely packed crests, affecting the efficiency of ATP production [31,32,33].

Early studies provided evidence that PrPc is highly expressed within human GBM cell lines [34,35], while recent evidence indicates that PrPc enhances the expression of a number of genes which are key in the onset and progression of GBM including a profound impact on mitochondrial turnover [17]. The role of PrPc in GBM is remarkable and it is likely to operate upstream in controlling the mitochondrial status.

The dual effects of mTOR-dependent autophagy activation provided in the present study (mitochondria status and prion protein) in two GBM cell lines are indeed very much connected. In fact, the occurrence of mitochondrial alterations is dramatic in the presence of high levels of prion protein and abnormally high prion protein produces a defect in mitochondrial fission/mitophagy, as recently shown by Li and coll. [17].

This is likely to explain why accumulation of altered mitochondria is considered a key factor in prion disease pathogenesis [17]. Remarkably, this is specifically induced by a defect in Pink1/PARK-induced mitochondrial removal. In the present study we extended the evidence connecting PrPc and defective mitochondria to GBM cells. The study shows that such a mitochondrial defect can be corrected by reverting the Pink1/PARK defect. In fact, rapamycin improved mitochondrial status and increased Pink1/PARK levels, while occluding PrPc accumulation. This was concomitant with mTOR inhibition and autophagy activation. In fact, PARK overexpression was already shown to mitigate defective mitophagy when PrPc is overexpressed [17]. Thus, an autophagy-dependent defect in Pink1/PARK-mediated mitophagy is induced by PrPc [17].

The role of PrPc in the onset and progression of disease were already evidenced in recent studies as the effects of multiple mechanisms concerning the growth of various tumors, differentiation, and resistance to radio- and chemo- therapy [15,36,37,38,39,40,41]. The occurrence of PrPc in GBM is more and more evident and it may contribute to cancer invasion and poor prognosis. It is remarkable that, according to Corsaro and coll. [41], PrPc expression in GBM may be responsible for promoting stemness, which in turn relates to tumor aggression and relapse. In fact, within GBM glioblastoma cancer stem cells express the highest level of PrPc in the tumor. These cells form typical neurospheres and possess remarkable proliferation rate and a loss of differentiation [15,41,42]. Conversely, suppression of PrPc expression produces a slower rate of proliferation along with cell differentiation and suppression of stem cell markers. All this evidence indicates that the expression of PrPc is key in the neurobiology of GBM and it seems to impact disease prognosis. The present study demonstrates the tighten relationship between autophagy, PrPc expression and mitochondrial status. The dual effects (mitochondrial status and PrPc expression) being scrutinized in the present research work using different GBM cell lines are likely to operate in symbiosis to transfer the impact of autophagy in the neurobiology of GBM. In fact, PrPc and mitochondria strongly impact cell proliferation. Thus, the autophagy dependent expression of proteins which promote mitochondrial fission is likely to strongly impact the course of GBM.

## 5. Conclusions

The present study investigates in different GBM cell lines whether mitochondrial impairment along with prion protein accumulation and the block of autophagy flux may all be reverted, within the same experimental settings, by simply removing an excess of mTOR activity. The present study measures whether, long-lasting effects produced by rapamycin concomitantly impact the following: (i) autophagy activation; (ii) prion protein clearance; (iii) improved mitochondrial status. These phenomena were related to the cytofluorimetry of GBM cells to assess whether a percentage of these cells under mTOR inhibition shifts from a proliferating into a quiescent phase. This study is seminal to correlate the accumulation of the GBM promoting protein PrPc with defective mitochondrial dynamics and relented autophagy flux, as well as whether an empowered autophagy flux fuels mitochondrial dynamics to shift GBM cells towards a non-proliferative phase in the cell cycle. A short-lasting exposure to rapamycin (24 h) produces long-lasting effects on the autophagy flux, prion protein clearance, mitochondrial status, and suppression of cell proliferation.

This study represents a further step to identify how the autophagy machinery interacts with the biology of GBM.

## Data Availability

The data that supports the findings of this study are available from the corresponding author upon reasonable request.

## Supplementary Materials

The following supporting information can be downloaded at: https://www.mdpi.com/article/10.3390/cells12020221/s1, Supplementary Figure S1: Rapamycin produces a long-lasting inhibition of mTOR activity in A172 cells. Supplementary Figure S2: Rapamycin produces a long-lasting decrease of P70S6K immuno-fluorescence in U87MG cells. Supplementary Figure S3: Rapamycin produces a long-lasting decrease of P70S6K immuno-fluorescence in A172 cells. Supplementary Figure S4: Rapamycin treatment increases the autophagy flux at 24 h in A172 cells. Supplementary Figure S5: Rapamycin exerts long-lasting effects on the autophagy flux at 4 days from its withdrawal in A172 cells. Supplementary Figure S6. Rapamycin induces long-lasting effects on the autophagy flux at 7 days from its withdrawal in A172 cells. Supplementary Figure S7. Rapamycin effects on the autophagy flux at 14 days in A172 cells. Supplementary Figure S8. Rapamycin increases immuno-fluorescent puncta positive for LC3 + Cat D in A172 cells. Supplementary Figure S9. Rapamycin induces both autophagosomes and lysosomes and increases the merging between these compartments in U87MG cells. Supplementary Figure S10. Rapamycin induces both autophagosomes and lysosomes and increases the merging between these compartments in A172 cells. Supplementary Figure S11. Rapamycin decreases PrPc protein in the cytoplasm of A172 cells. Supplementary Figure S12. Rapamycin decreases PrPc and PrPsc-like proteins in the cytoplasm of A172 cells. Supplementary Figure S13. Rapamycin persistently increases the MTR-G fluorescence in A172 cells. Supplementary Figure S14. Rapamycin persistently increases the number of mitochondria in A172 cells. Supplementary Figure S15. Rapamycin long-lasting rescues altered mitochondria in A172 cells. Supplementary Figure S16. Rapamycin increases the merging of MTR-G with LC3 in A172 cells. Supplementary Figure S17. Rapamycin increases PARK immuno-gold particles in U87MG cells. Supplementary Figure S18. Rapamycin increases Pink1 immuno-gold particles within mitochondria of U87MG cells. Supplementary Figure S19. Rapamycin increases the merging of parkin (PARK) and Pink1 immunofluorescence in A172 cells. Supplementary Figure S20. Rapamycin increases PARK immuno-gold particles in A172 cells. Supplementary Figure S21. Rapamycin increases Pink1 immuno-gold particles within mitochondria of A172 cells. Supplementary Figure S22. Rapamycin increases the merging of MTR-G with BNIP3 in A172 cells. Supplementary Figure S23. Rapamycin increases the fission protein marker Fis1. Supplementary Figure S24. Rapamycin increases the fission protein marker DRP1. Supplementary Figure S25. Rapamycin increases immunofluorescence for Fis1 and DRP1 in A172 cells.
