# Supplementary material for: Autophagy Activation Associates with Suppression of Prion Protein and Improved Mitochondrial Status in Glioblastoma Cells
Source: Cells. 2023 Jan 4;12(2):221. doi: 10.3390/cells12020221 (PMC9857229; doi:10.3390/cells12020221)
Supplement: Supplementary file 1 [file cells-12-00221-s001.zip › cells-1847005-supplementary.pdf]

Supplementary Figures

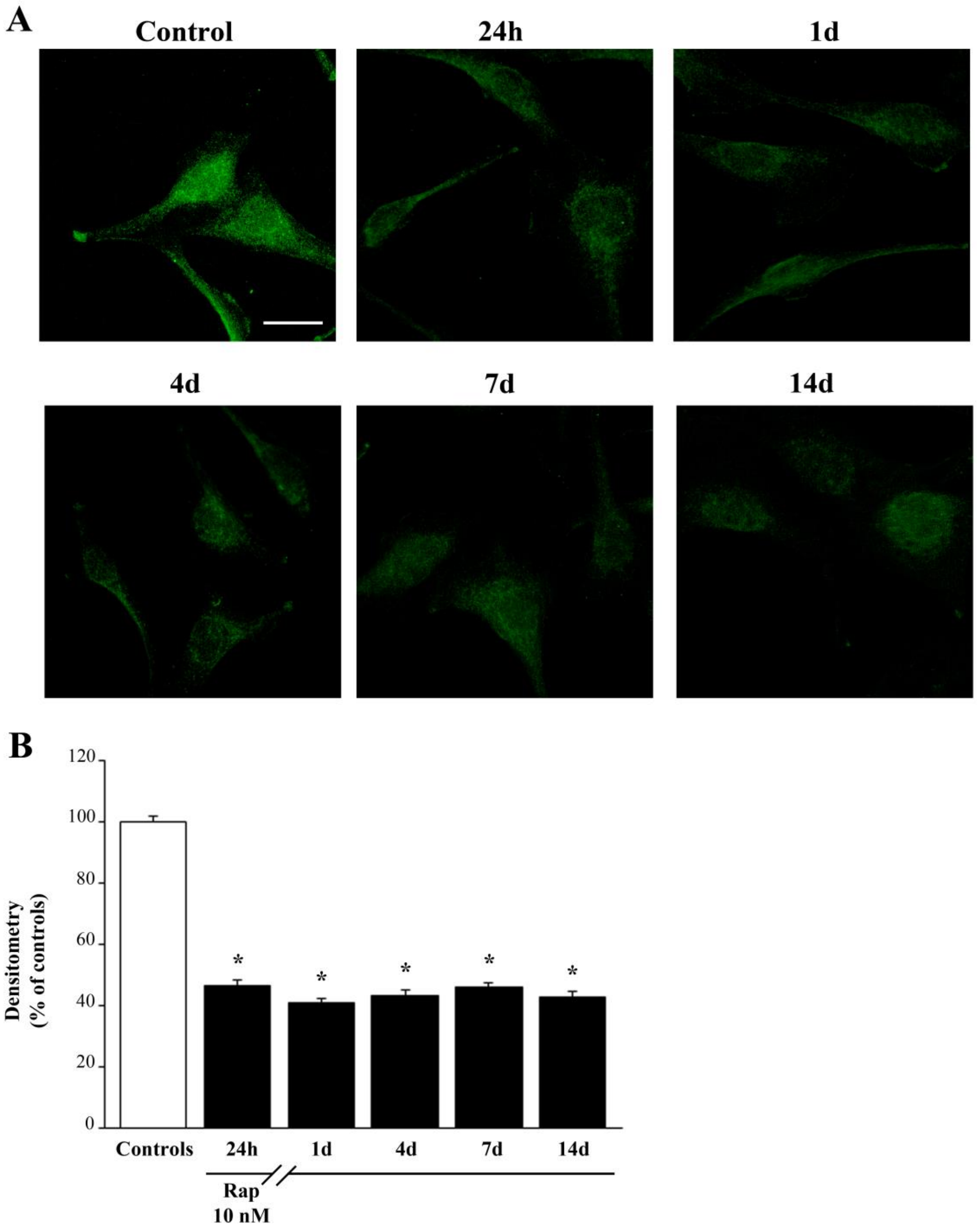

Figure S1. Rapamycin produces a long-lasting inhibition of mTOR activity in A172 cells. (**A**) Representative pictures showing the marked and long-lasting decrease of the immuno-fluorescence of the downstream enzymatic product of mTOR, PS6RP. The graph in (**B**), which reports the densitometry of the PS6RP immuno-fluorescence, demonstrates that rapamycin induces a massive mTOR inhibition, which lasts at least 14 days following rapamycin withdrawal. Data are given as the mean percentage±S.E.M. of optical density measured in 100 cells per group (assuming controls as 100%). *$p<0.05$ compared with controls. Scale bar=16 μm.

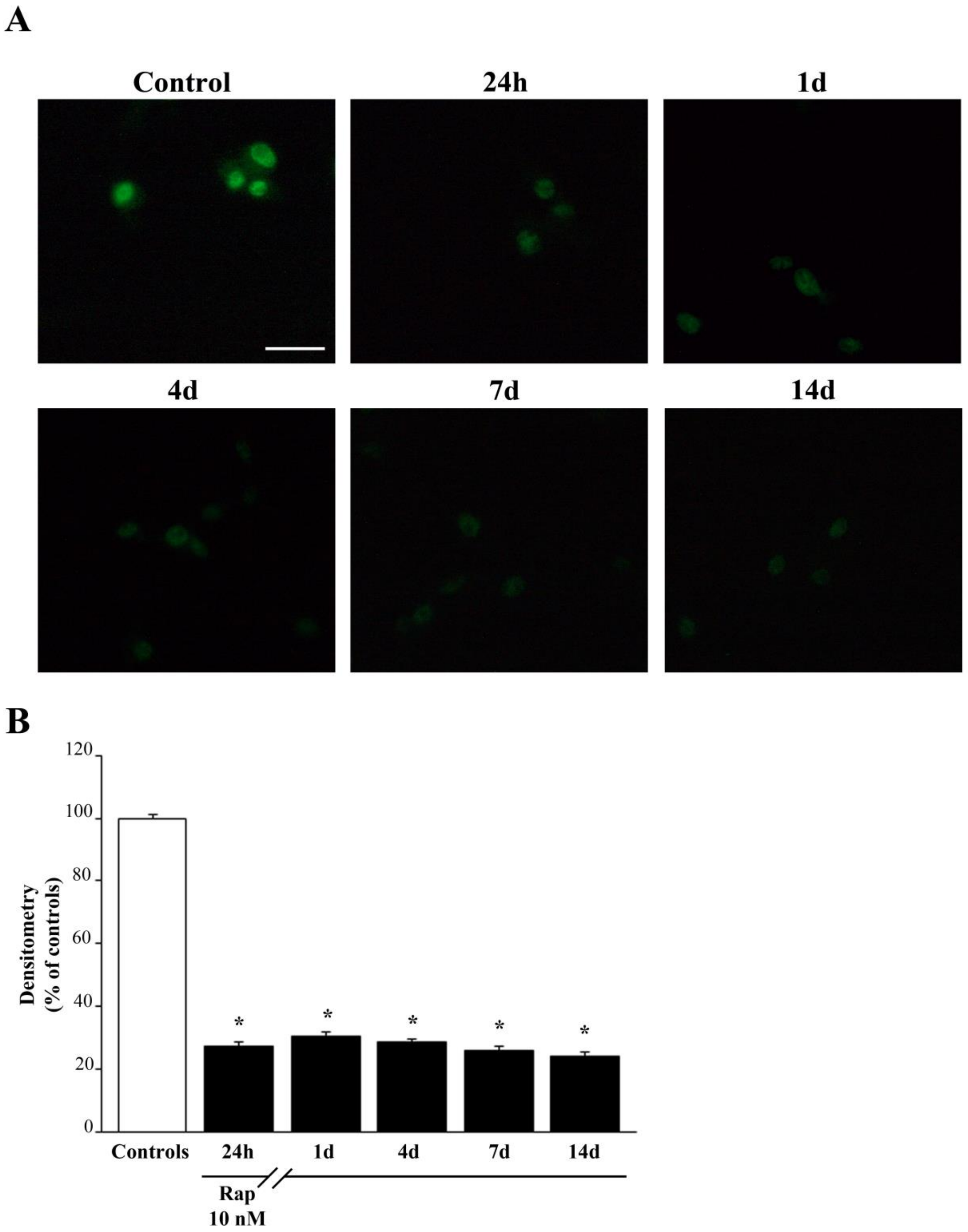

Figure S2. Rapamycin produces a long-lasting decrease of P70S6K immuno-fluorescence in U87MG cells. (**A**) Representative immuno-fluorescence of the P70S6K protein, the direct mTOR substrate, shows a marked and long-lasting decrease of the P70S6K staining produced by rapamycin. This is confirmed by the graph in (**B**), which reports the densitometry of the P70S6K immuno-fluorescence. Data are given as the mean percentage±S.E.M. of optical density measured in 100 cells per group (assuming controls as 100%). *$p<0.05$ compared with controls. Scale bar=60 μm.

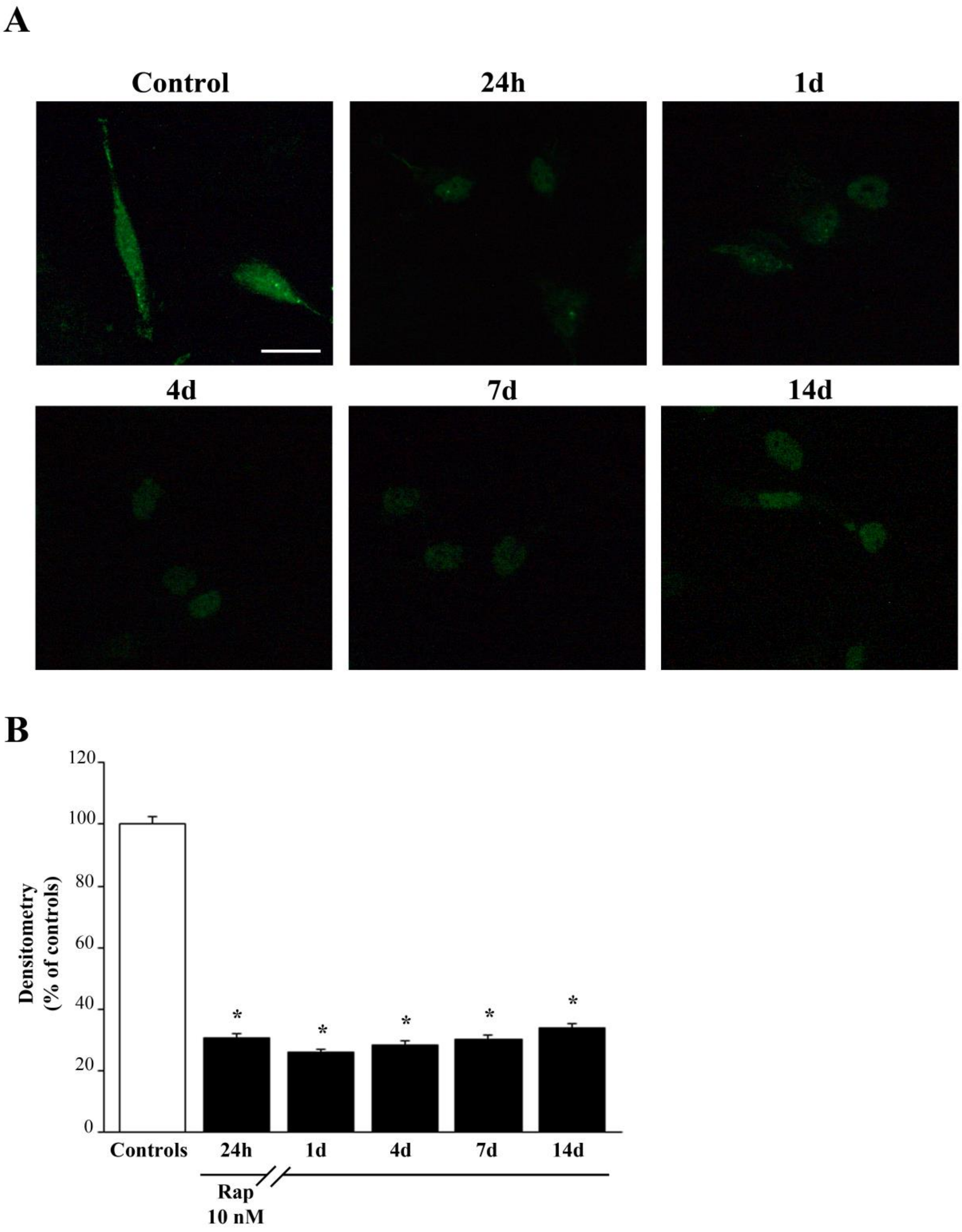

Figure S3. Rapamycin produces a long-lasting decrease of P70S6K immuno-fluorescence in A172 cells. (**A**) Representative immuno-fluorescence of the P70S6K protein, the direct mTOR substrate, shows a marked and long-lasting decrease of the P70S6K staining produced by rapamycin. This is confirmed by the graph in (**B**), which reports the densitometry of the P70S6K immuno-fluorescence. Data are given as the mean percentage±S.E.M. of optical density measured in 100 cells per group (assuming controls as 100%). *$p<0.05$ compared with controls. Scale bar=34 μm.

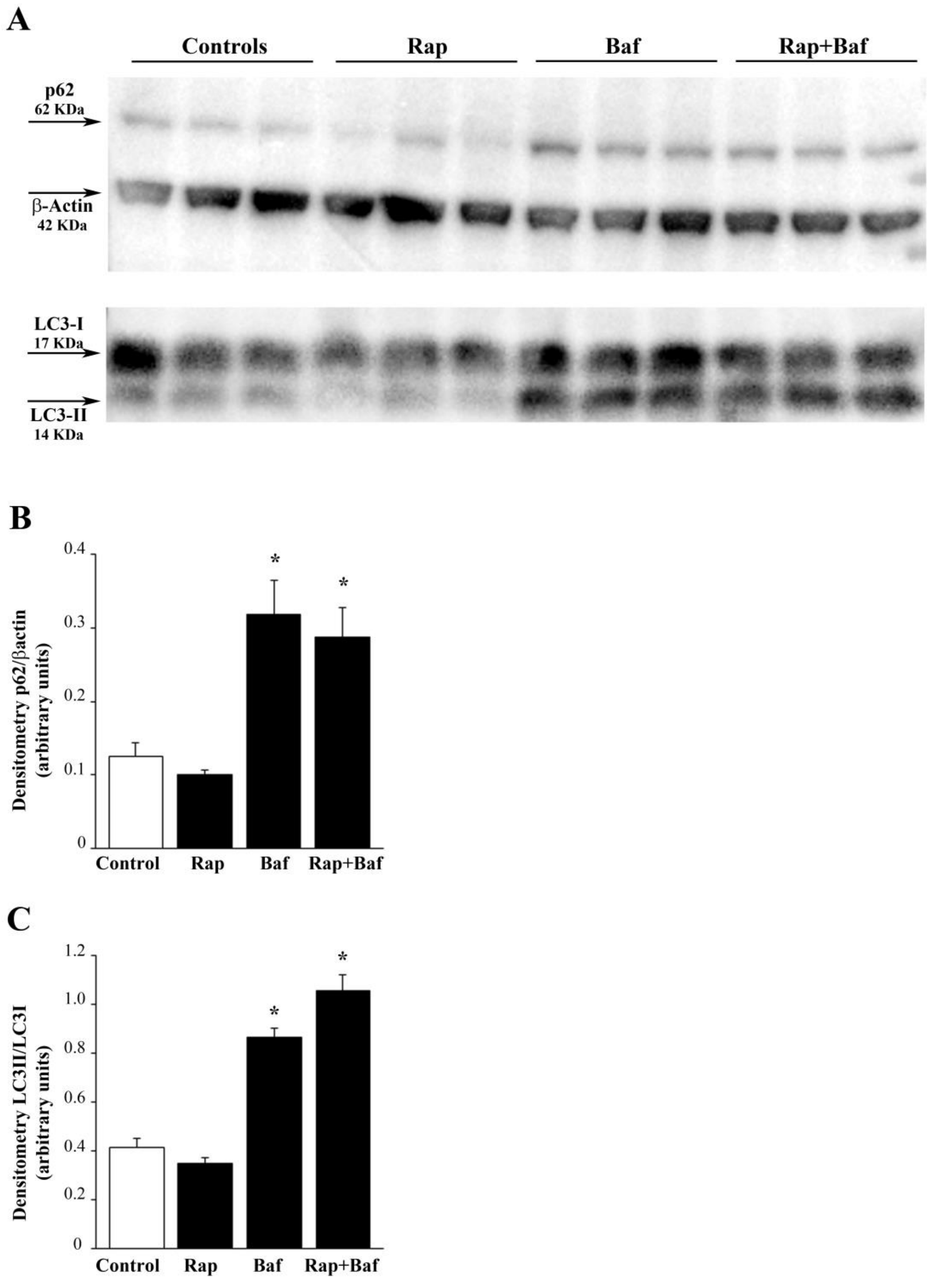

Figure S4. Rapamycin treatment increases the autophagy flux at 24h in A172 cells. (**A**) Western blot analysis of p62 and LC3-II in U87MG cells treated with 10 nM rapamycin for 24h; in some samples bafilomycin A1 (100 nM) was added during the last 3h in both untreated and rapamycin-treated cultures. Densitometric analysis of the level of p62 compared with the housekeeping β-actin (**B**) and LC3-II compared with LC3-I (**C**) are shown. Values are given as the mean±S.E.M. from three samples per experimental group. *p<0.05 compared with controls.

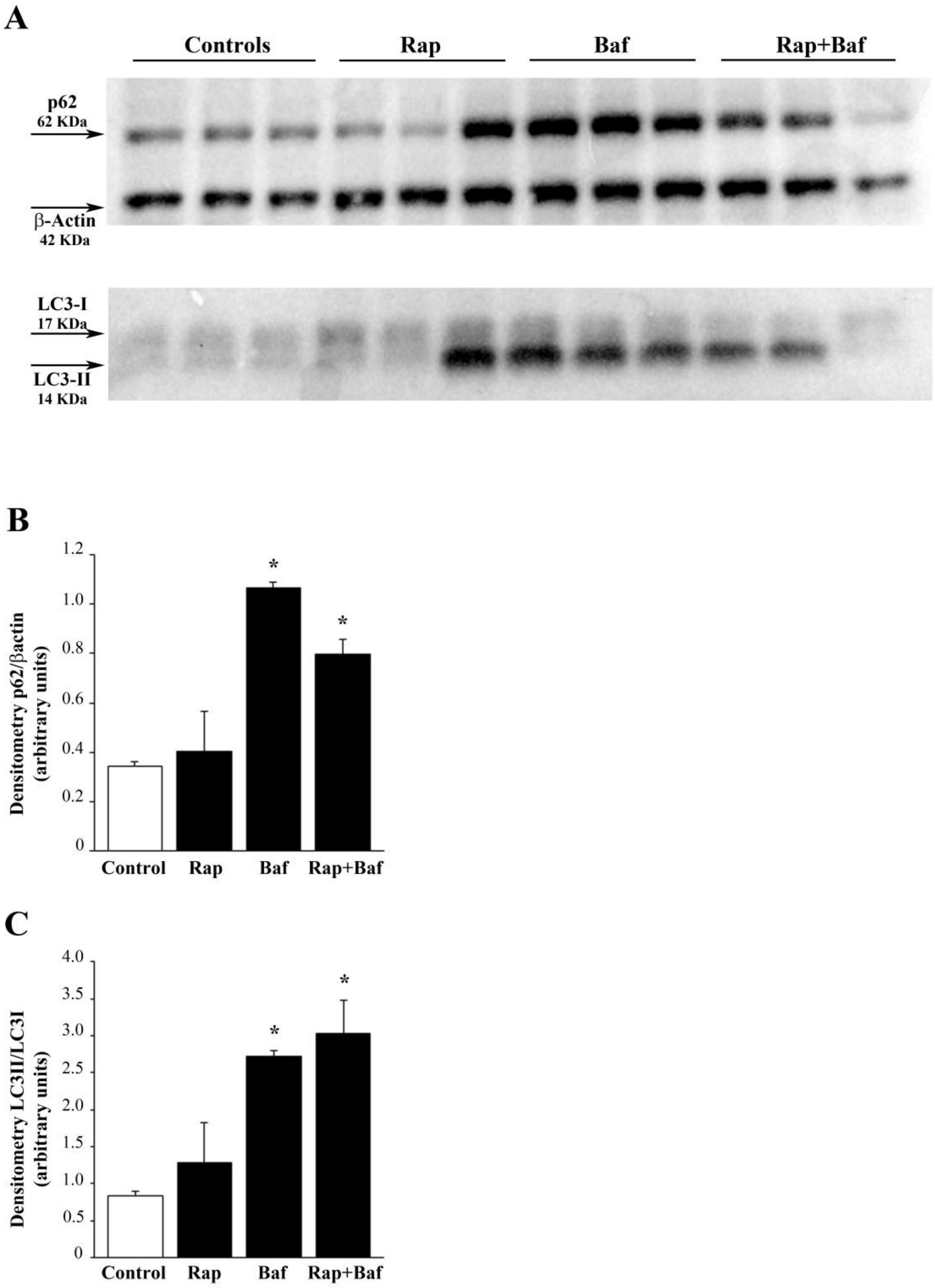

Figure S5. Rapamycin exerts long-lasting effects on the autophagy flux at 4 days from its withdrawal in A172 cells. (**A**) A172 cells were treated with 10 nM rapamycin and western blot analysis of p62 and LC3-II was performed 4 days after its removal; in some samples bafilomycin (100 nM) was added during the last 3h in both untreated and rapamycin-treated cultures. Densitometric analysis of the level of p62 compared with the housekeeping β-actin (**B**) and LC3-II compared with LC3-I (**C**) are shown. Values are given as the mean±S.E.M. from three samples per experimental group. *$p < 0.05$ compared with controls.

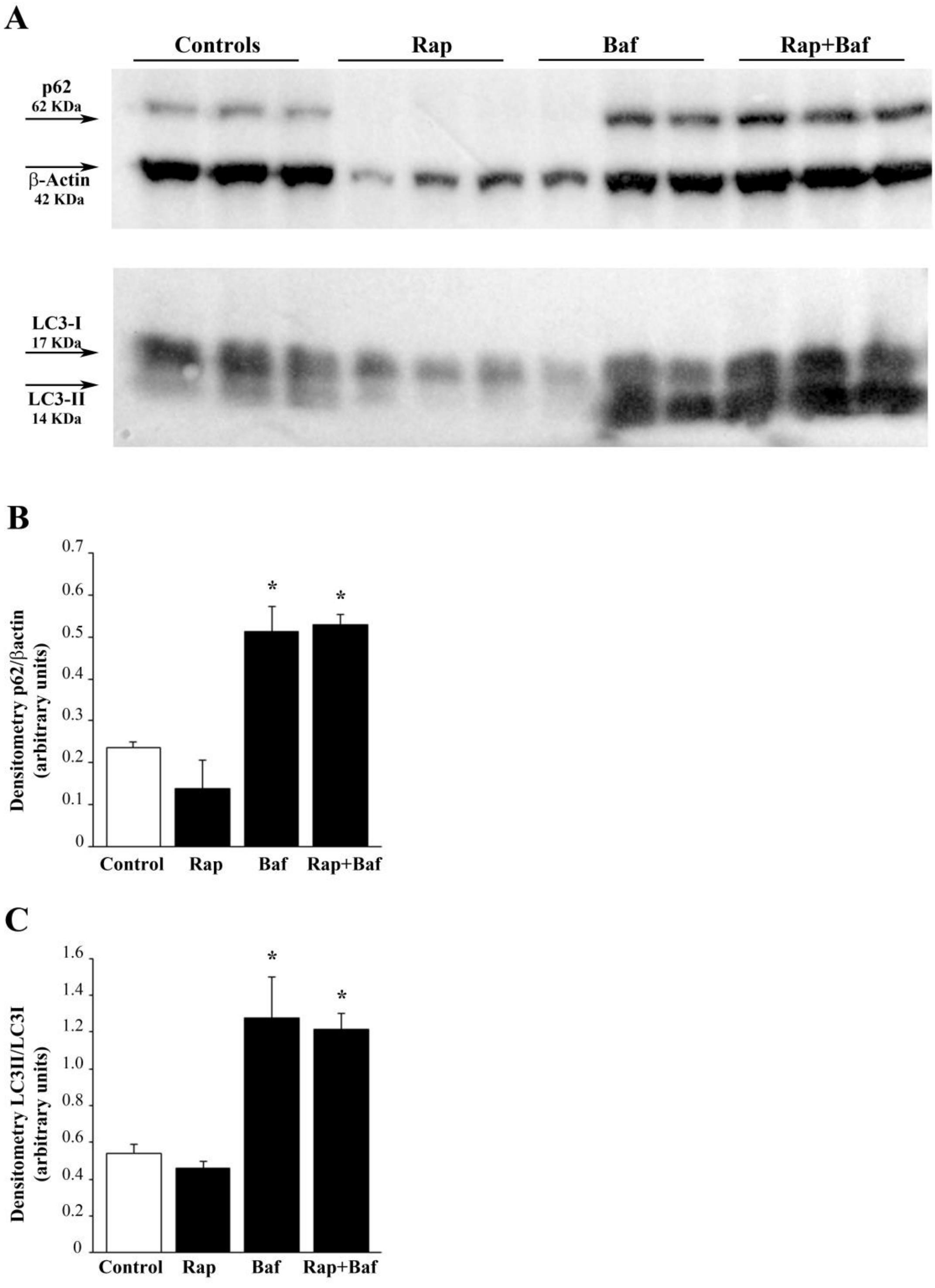

Figure S6. Rapamycin induces long-lasting effects on the autophagy flux at 7 days from its withdrawal in A172 cells. (**A**) A172 cells were treated with 10 nM rapamycin and western blot analysis of p62 and LC3-II was performed 7 days after its removal; in some samples bafilomycin (100 nM) was added during the last 3 h in both untreated and rapamycin-treated cultures. Densitometric analysis of the level of p62 compared with the housekeeping β-actin (**B**) and LC3-II compared with LC3-I (**C**) are shown. Values are given as the mean±S.E.M. from three samples per experimental group. *$p < 0.05$ compared with controls.

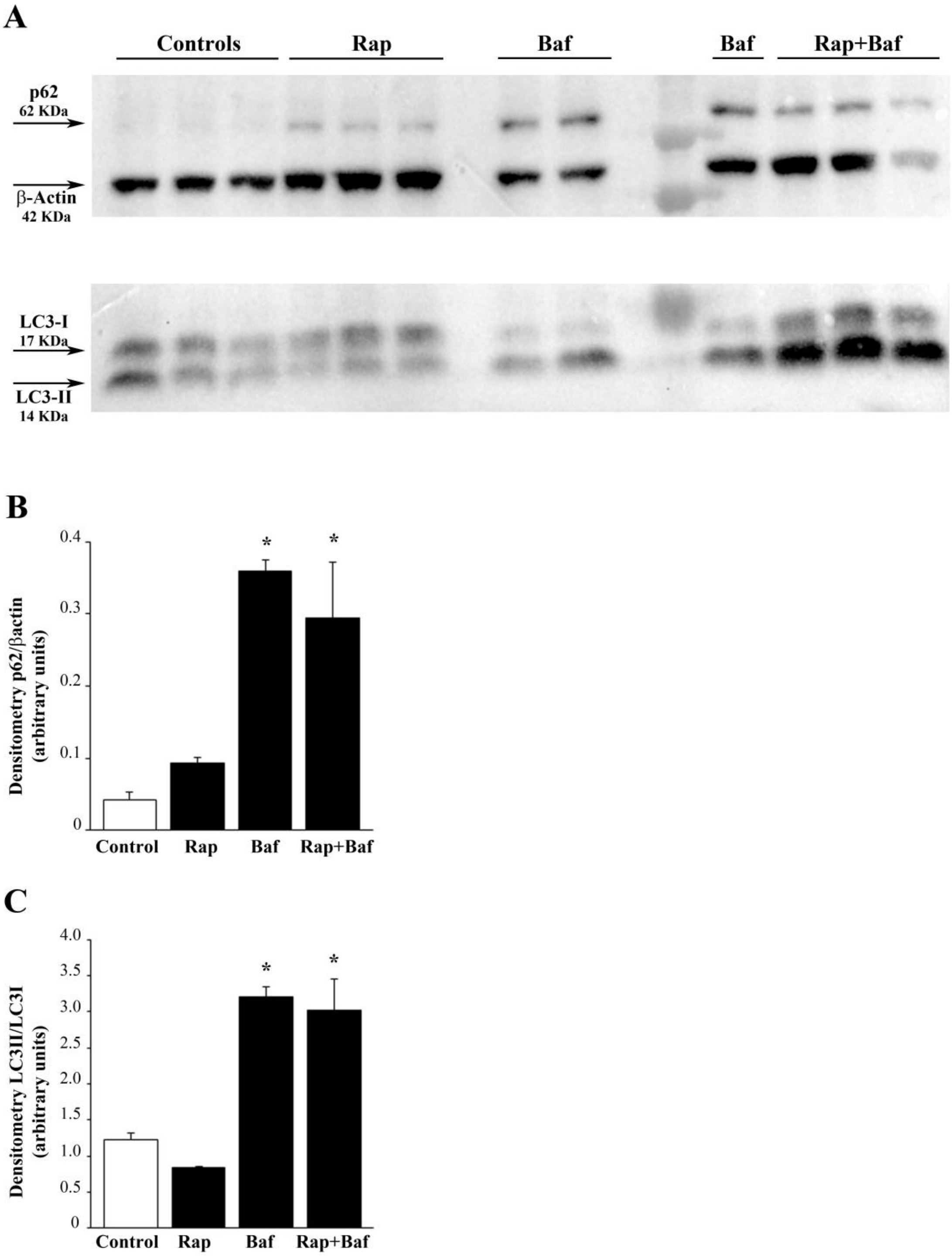

Figure S7. Rapamycin effects on the autophagy flux at 14 days in A172 cells. (**A**) A172 cells were treated with 10 nM rapamycin and western blot analysis of p62 and LC3-II was performed 14 days after its removal; in some samples bafilomycin (100 nM) was added during the last 3 h in both untreated and rapamycin-treated cultures. Densitometric analysis of the level of p62 compared with the house-keeping β-actin (**B**) and LC3-II compared with LC3-I (**C**) are shown. Values are given as the mean±S.E.M. from three samples per experimental group. *$p < 0.05$ compared with controls.

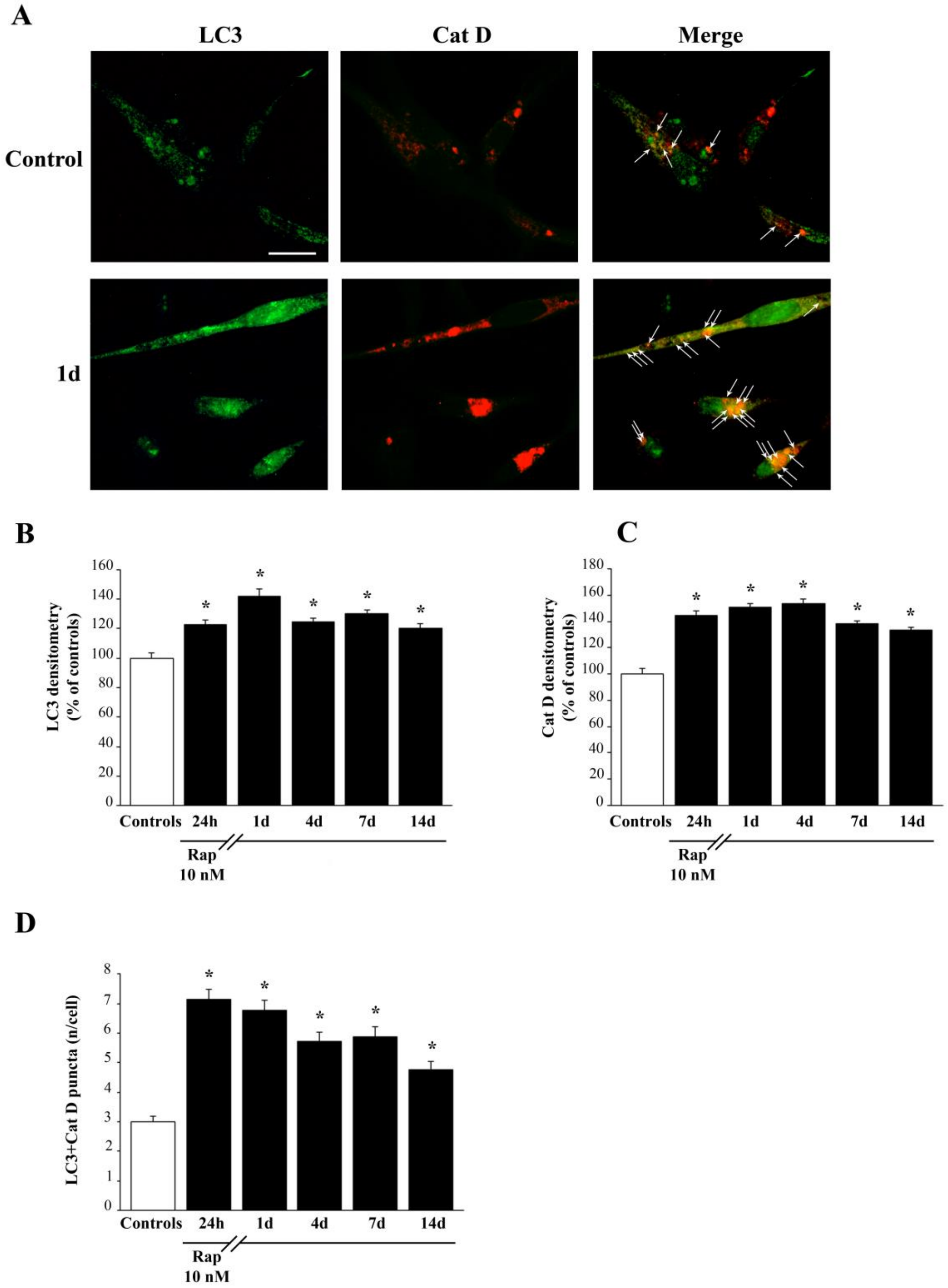

Figure S8. Rapamycin increases immuno-fluorescent puncta positive for LC3+Cat D in A172 cells. (**A**) Representative immuno-fluorescent pictures of the autophagy markers LC3 and Cat D and their merging. Arrows point to merging (yellow) puncta. Graphs report: (**B**) LC3 fluorescent density, (**C**) Cat D fluorescent density, and (**D**) LC3+Cat D merging puncta. Counts report means ±S.E.M. from three independent experiments. *$p<0.05$ compared with controls. Scale bar=14 μm.

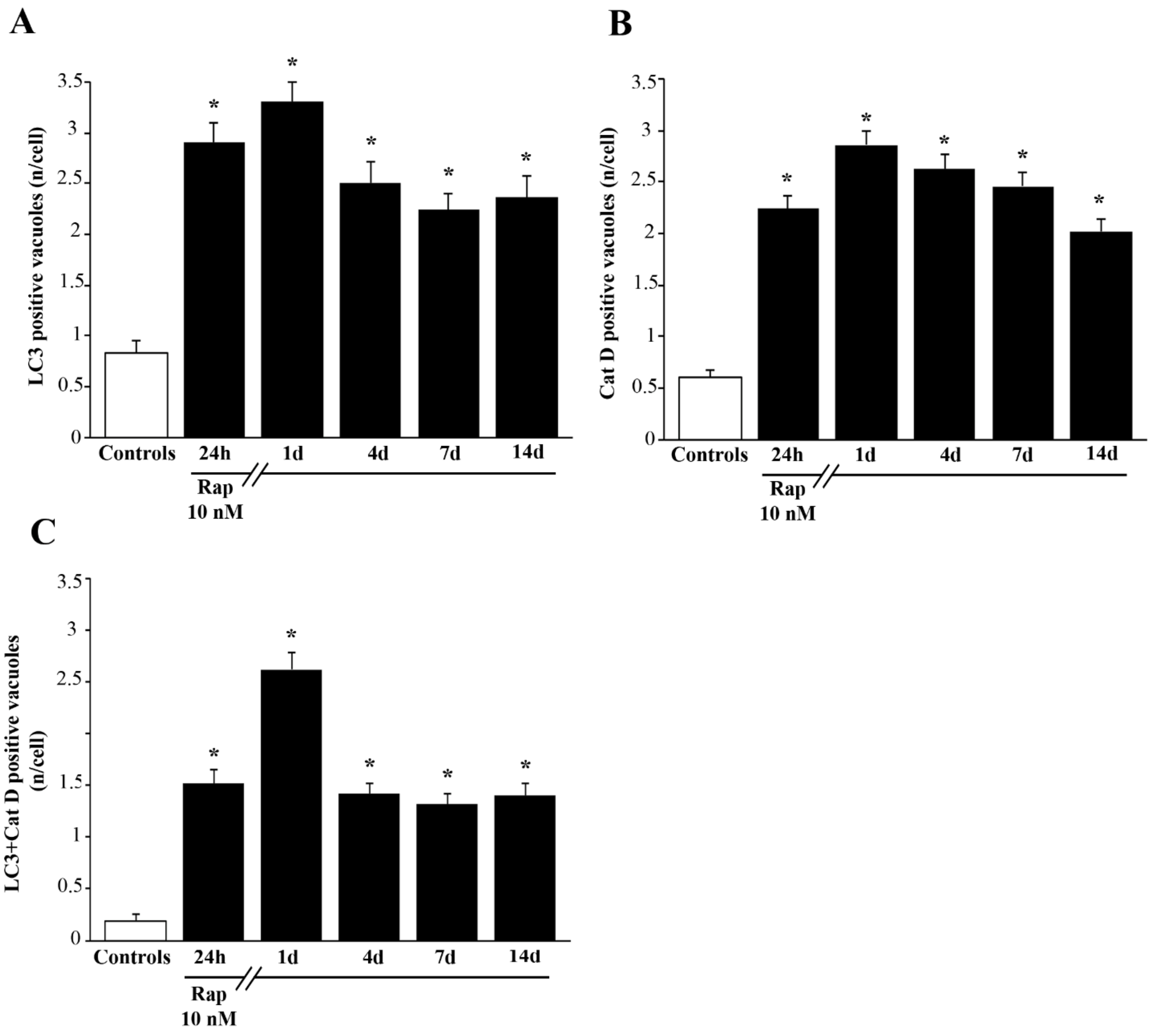

Figure S9. Rapamycin induces both autophagosomes and lysosomes and increases the merging between these compartments in U87MG cells. Graphs report ultrastructural morphometry of (**A**) the number of LC3 positive vacuoles (autophagosomes), (**B**) the number of Cat D positive vacuoles (lysosomes) and (**C**) the number of LC3+Cat D positive vacuoles (autophagolysosomes) in U87MG cells in control conditions and at various times after rapamycin treatment. Values are given as the mean±S.E.M. from N=50 cells per group. *$p<0.05$ compared with controls.

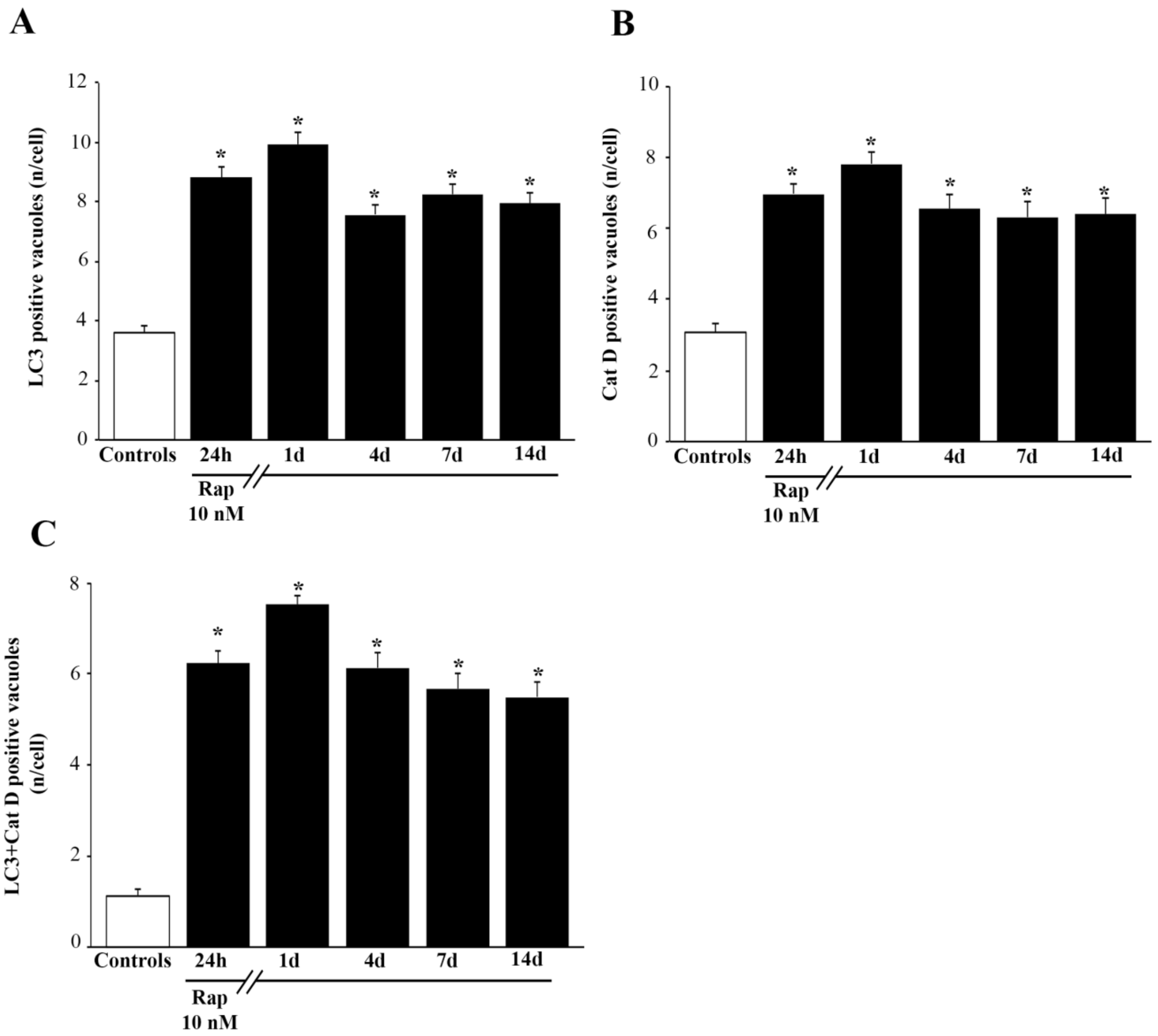

Figure S10. Rapamycin induces both autophagosomes and lysosomes and increases the merging between these compartments in A172 cells. Graphs report ultrastructural morphometry of (**A**) the number of LC3 positive vacuoles (autophagosomes), (**B**) the number of Cat D positive vacuoles (lysosomes) and (**C**) the number of LC3+Cat D positive vacuoles (autophagolysosomes) in A172 cells in control conditions and at various times after rapamycin treatment. Values are given as the mean±S.E.M. from N=50 cells per group. *$p<0.05$ compared with controls.

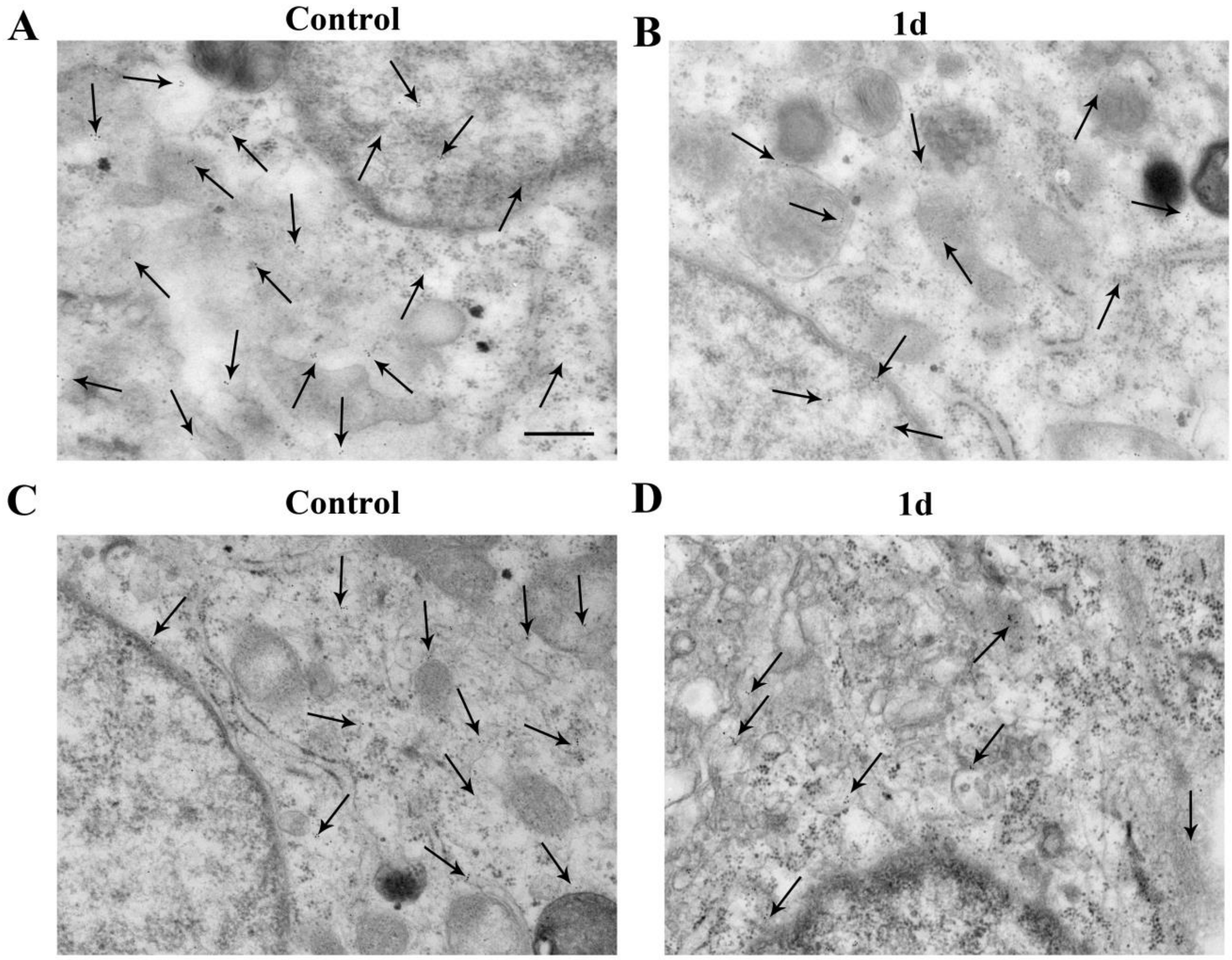

Figure S11. Rapamycin decreases PrPc protein in the cytoplasm of A172 cells. Representative TEM micrographs showing a PrPc- or PrPsc-like stained control cell (**A** and **C**, respectively) and a cell following 1d of rapamycin withdrawal (**B** and **D**, respectively). Arrows point to PrPc/ PrPsc-like immuno-gold particles within the cytosol. Scale bar= 170 nm.

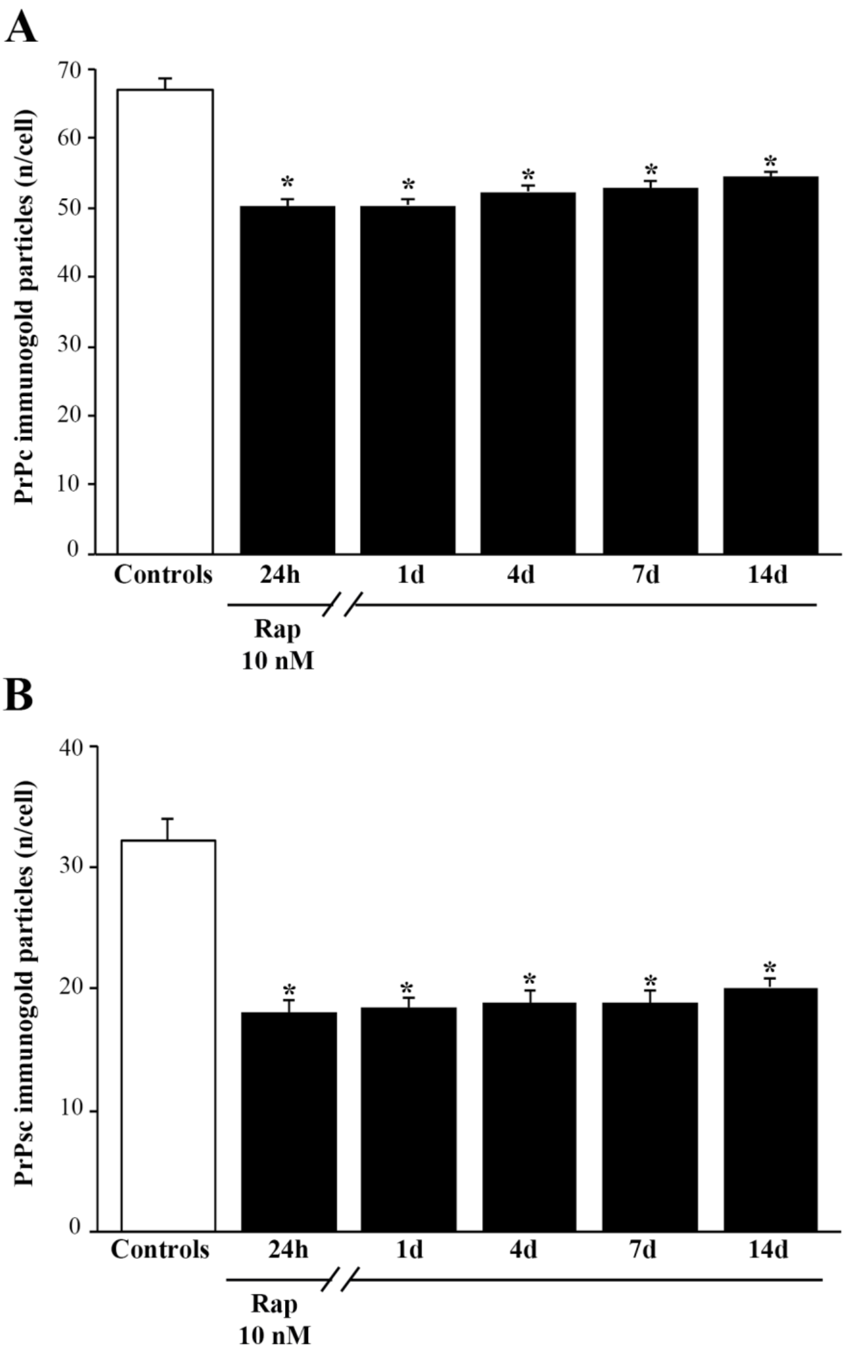

Figure S12. Rapamycin decreases PrPc and PrPsc-like proteins in the cytoplasm of A172 cells. Graphs report the quantitative ultrastructural morphometry for PrPc (**A**) and PrPsc-like (**B**) immuno-gold particles within the cytosol. Counts represent the mean±S.E.M from N=50 cells per group. *$p<0.05$ compared with control.

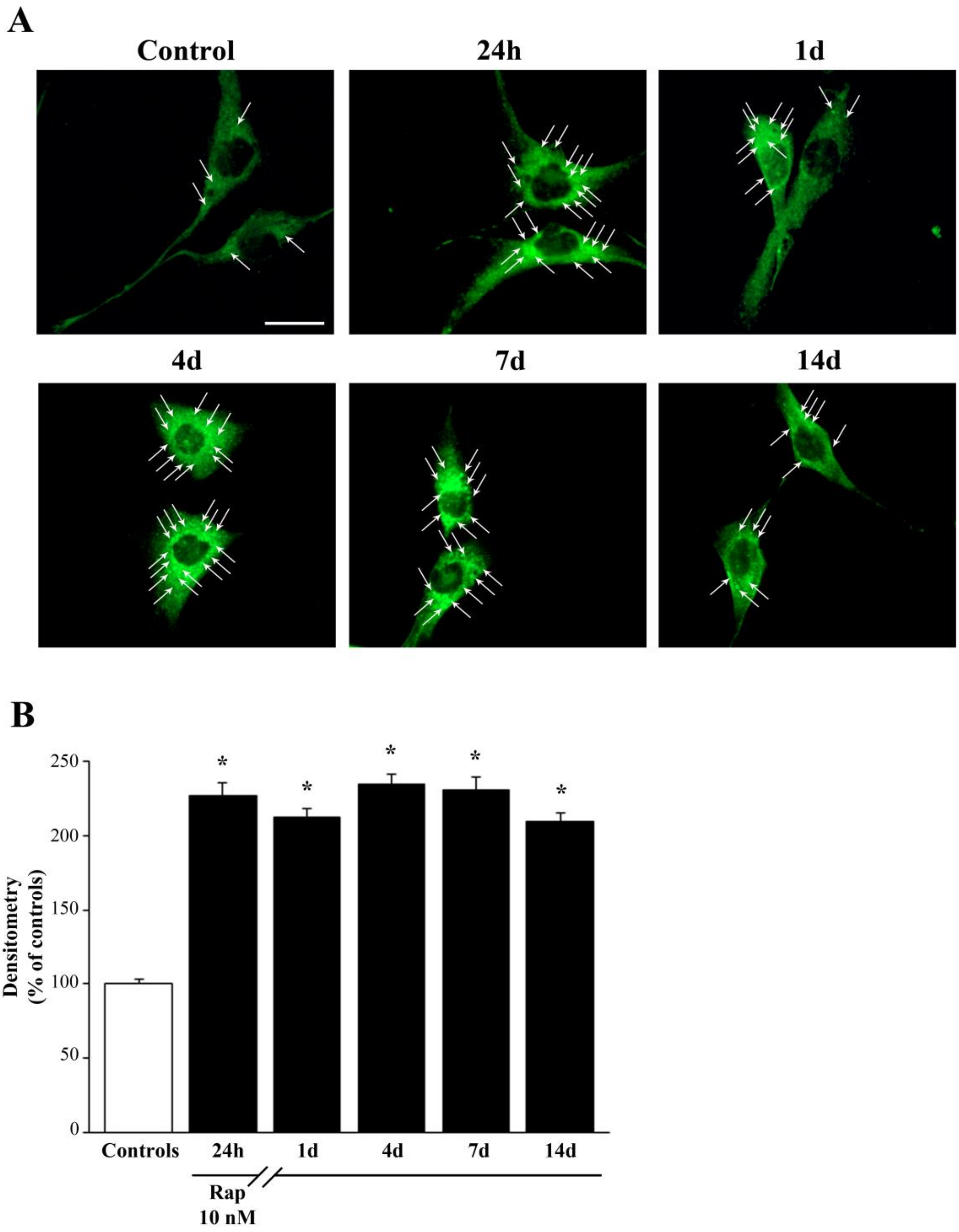

Figure S13. Rapamycin persistently increases the MTR-G fluorescence in A172 cells. (**A**) Representative pictures showing total mitochondria stained with MTR-G from controls, rapamycin-treated cells (10 nM) for 24h and after rapamycin withdrawal (up to 14d). Arrows indicate intense fluorescence within the cells. (**B**) Graph reports the increase in mitochondrial fluorescence induced by rapamycin, which persists unmodified for 14 days. Values are given as the mean±S.E.M from N=100 cells per group *$p<0.05$ compared with control. Scale bar=15 µm.

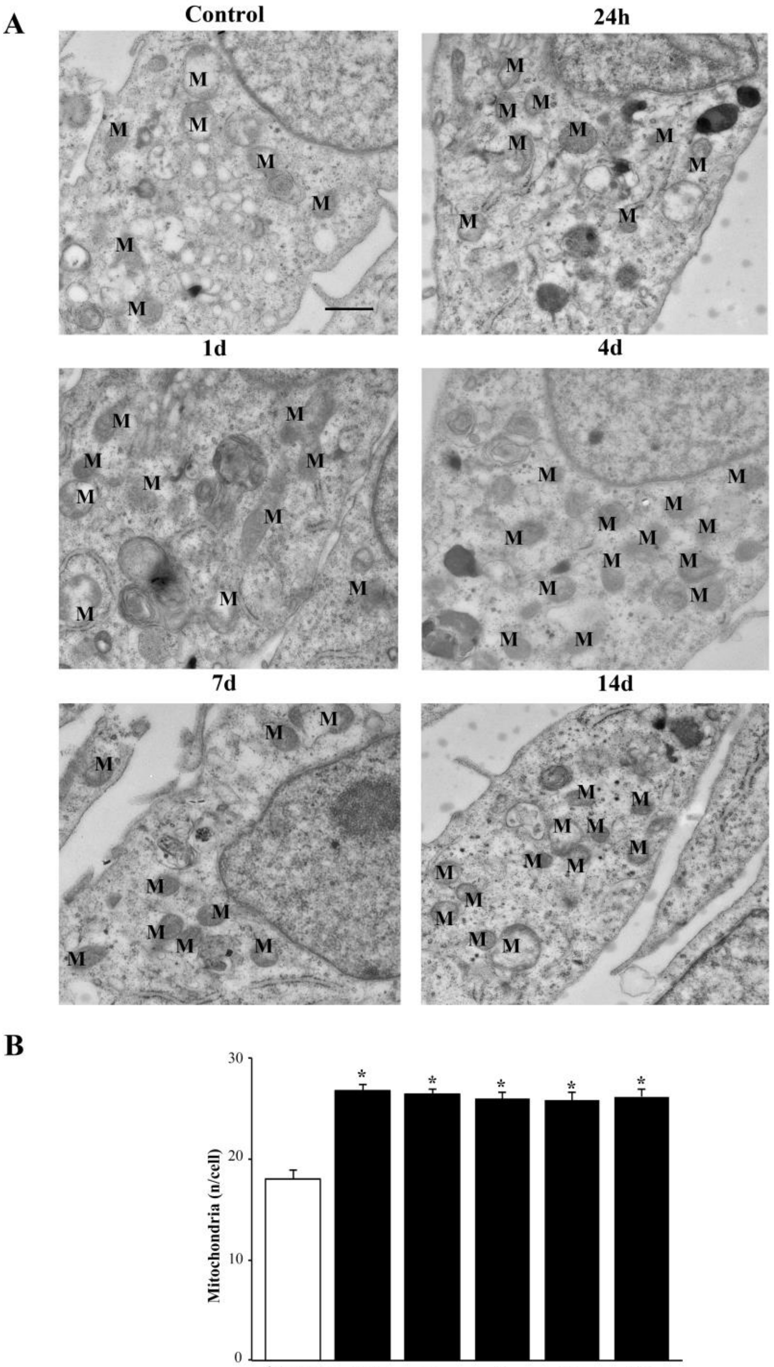

Figure S14. Rapamycin persistently increases the number of mitochondria in A172 cells. (**A**) Representative pictures at TEM show the increase in mitochondria (M) counted directly as specific organelles. (**B**) Graph reports the number of mitochondria per cell counted at TEM, which similarly persists at 14 days. Values are given as the mean±S.E.M from N=50 cells per group. *$p<0.05$ compared with control. Scale bar=260 nm.

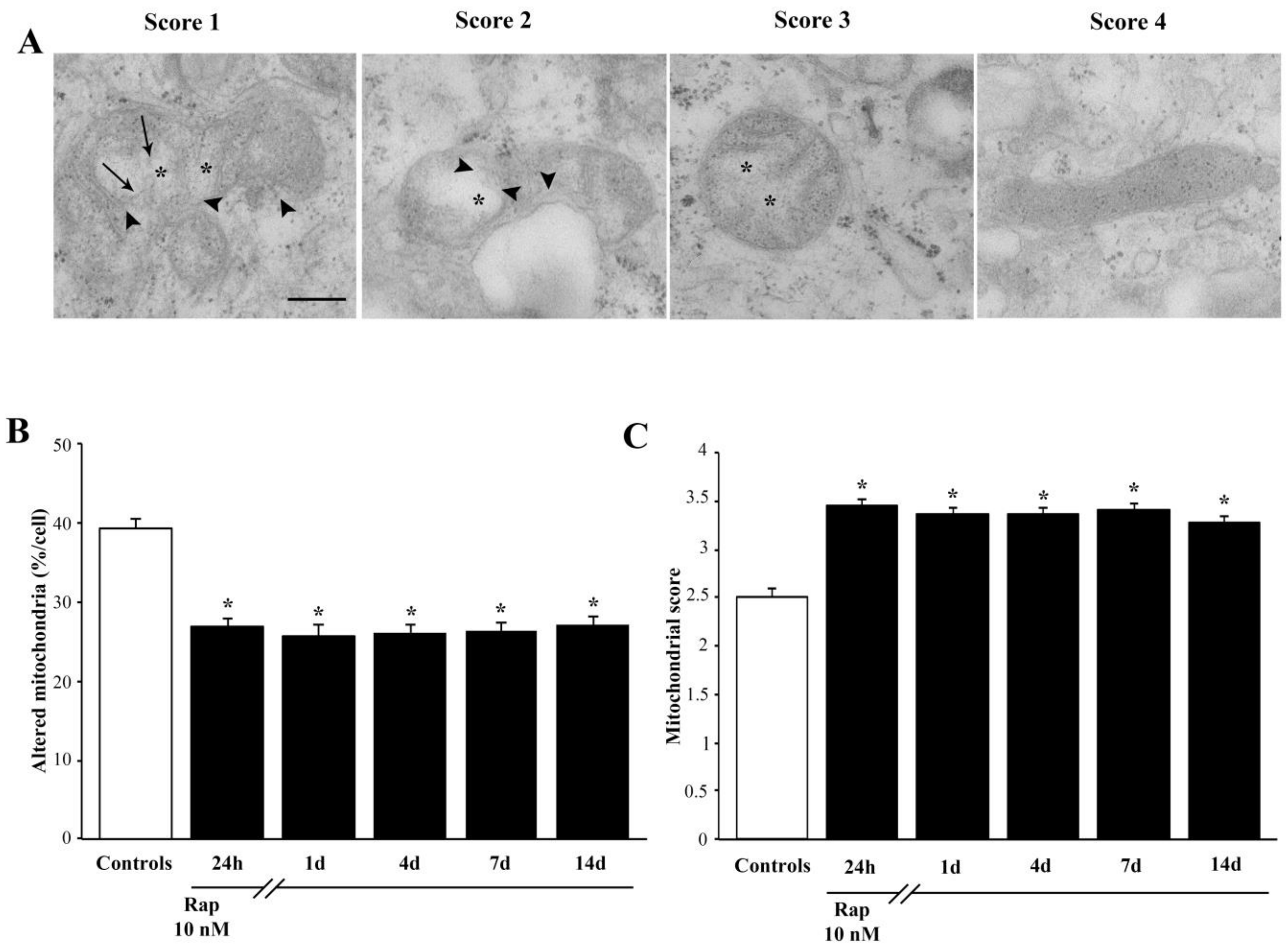

Figure S15. Rapamycin long-lasting rescues altered mitochondria in A172 cells. (**A**) Representative ultrastructural pictures of mitochondria, showing specific morphological features which are evaluated based on the modified scale from Flameng et al. 1980 [23] (see the main manuscript for the meaning of the score). Graph (**B**) reports the percentage of altered mitochondria (including all kinds of alterations). Graph (**C**) reports the average of mitochondrial score. Values are given as (**B**) the percentage±S.E.M from N=50 cells per group or (**C**) the mean mitochondrial morphological score±S.E.M calculated by averaging mitochondrial scores from at least 150 mitochondria from each experimental group. *$p<0.05$ compared with controls. Scale bar=140 nm.

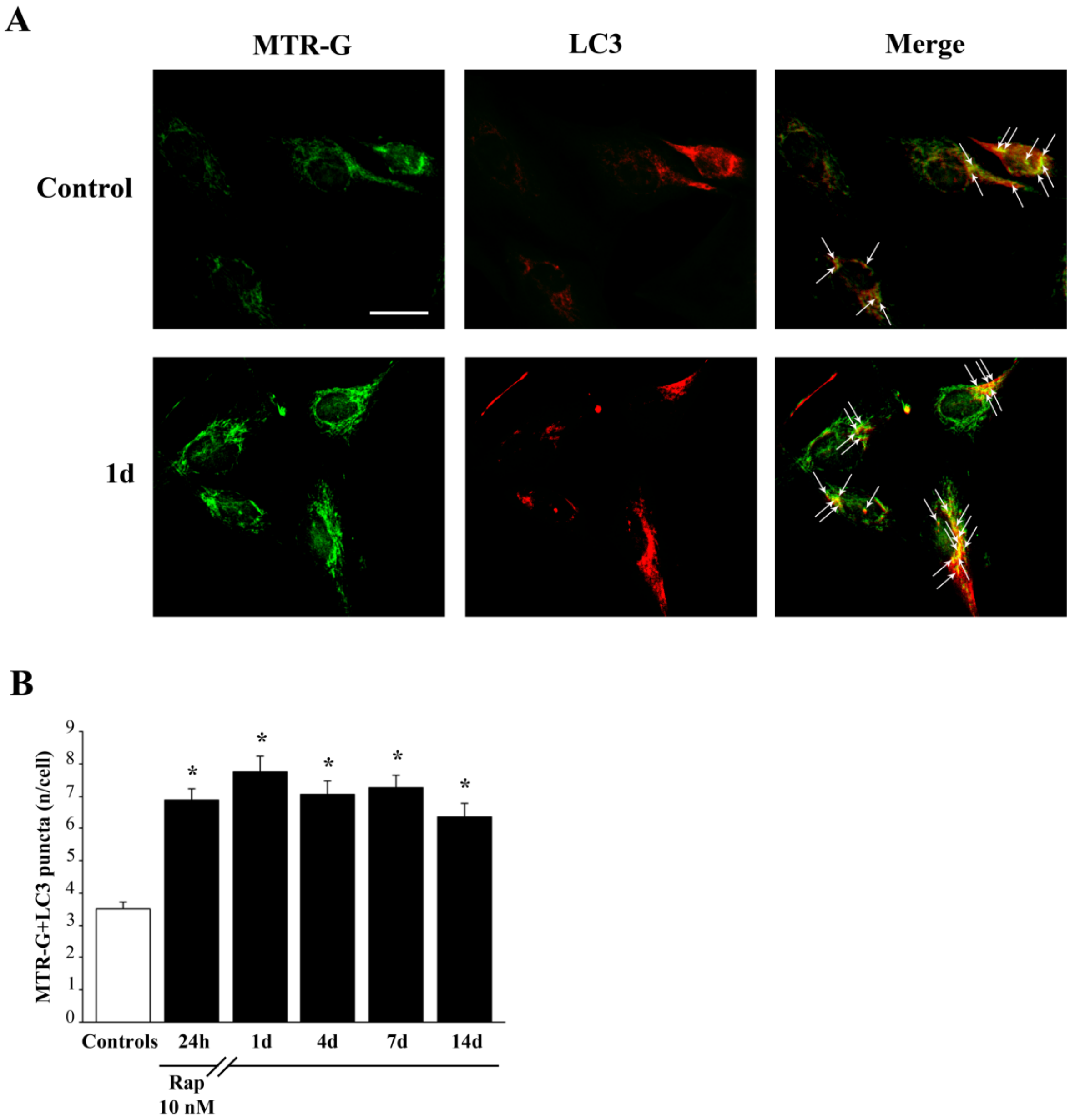

Figure S16. Rapamycin increases the merging of MTR-G with LC3 in A172 cells. (**A**) Representative pictures showing that rapamycin increases the MTR-G fluorescence along with LC3 immuno-fluorescence. Arrows point to merging (yellow) puncta. (**B**) Graph reports the rapamycin-induced increase in MTR-G+LC3 merging puncta, which persists up to 14 d. Data are given as the mean±S.E.M. from N=100 cell per group. *$p<0.05$ compared with controls. Scale bar=18 μm.

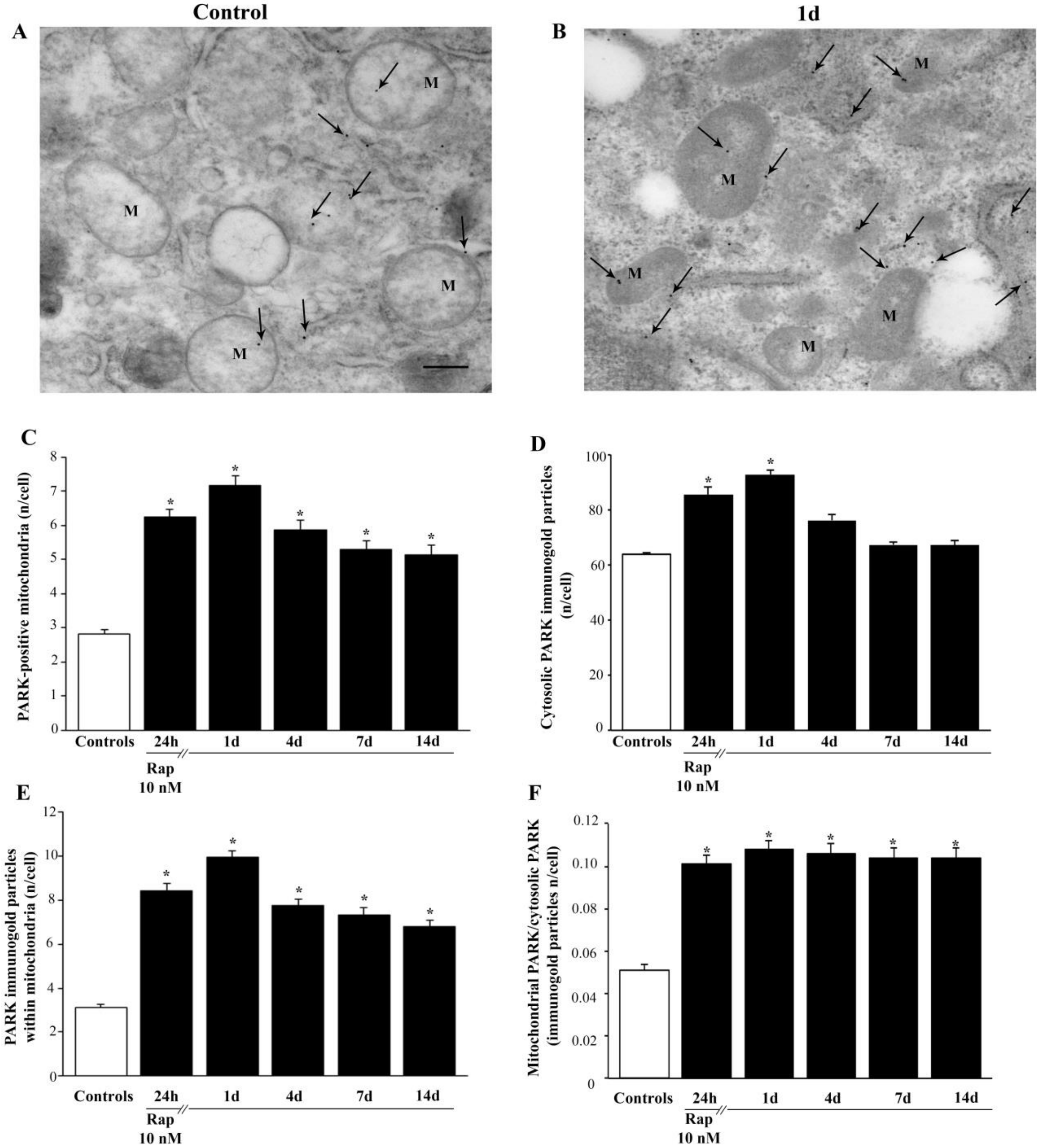

Figure S17. Rapamycin increases PARK immuno-gold particles in U87MG cells. Representative micrographs showing PARK immuno-gold in U87MG cells of control (**A**) and after 1d of rapamycin withdrawal (**B**). Graphs report: (**C**) PARK-positive mitochondria, (**D**) PARK immuno-gold particles within the cytosol, (**E**) PARK immuno-gold particles within mitochondria, and (**F**) the ratio of mitochondrial to cytosolic PARK particles. Values are given as the mean±S.E.M. from N=50 cells per group. *p<0.05 compared with controls. Scale bar=230 nm.

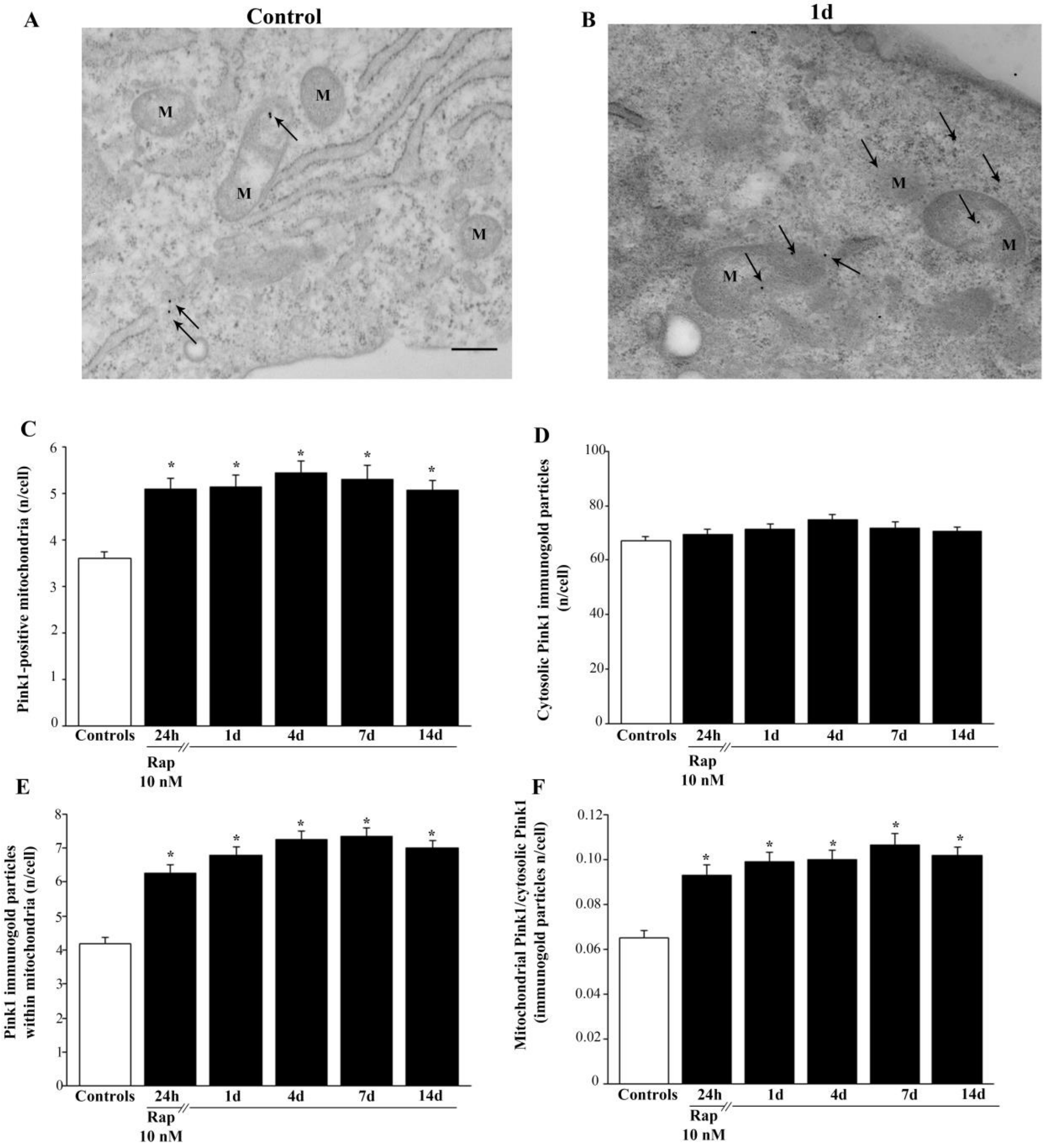

Figure S18. Rapamycin increases Pink1 immuno-gold particles within mitochondria of U87MG cells. Representative micrographs showing Pink1 immuno-gold in U87MG cells of control (**A**) and after 1d of rapamycin withdrawal (**B**). Graphs report: (**C**) Pink1-positive mitochondria, (**D**) Pink1 immuno-gold particles within the cytosol, (**E**) Pink1 immuno-gold particles within mitochondria, and (**F**) the ratio of mitochondrial to cytosolic Pink1 particles. Values are given as the mean±S.E.M. from N=50 cells per group. *p<0.05 compared with controls. Scale bar=230 nm.

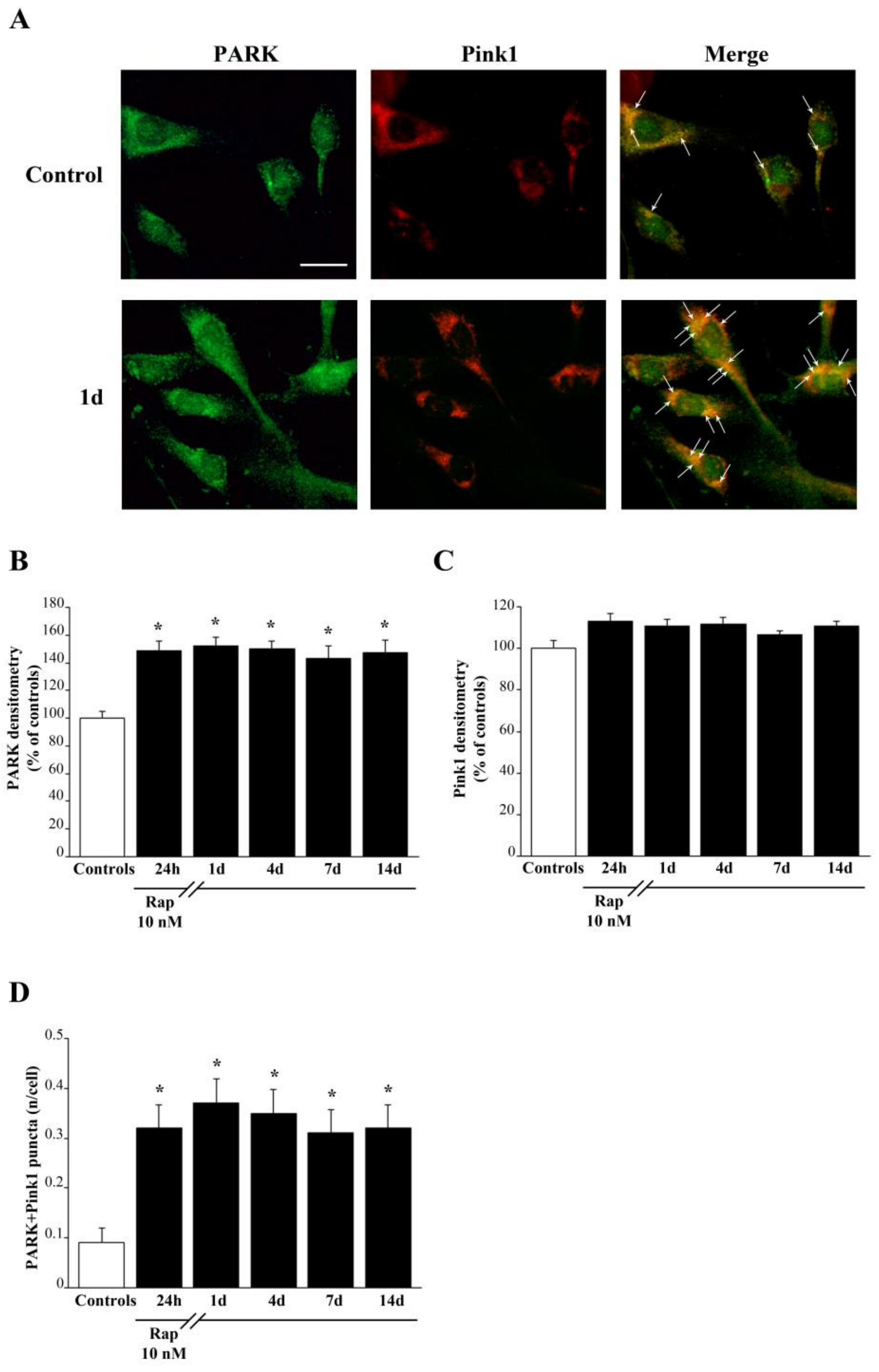

Figure S19. Rapamycin increases the merging of parkin (PARK) and Pink1 immunofluorescence in A172 cells. (**A**) Representative pictures showing the mitophagy markers PARK and Pink1 from control and rapamycin-treated cells (10 nM at 1 day). Arrows point to merging (yellow) puncta. The graph report: (**B**) a long-lasting significant increase of PARK immunofluorescence following rapamycin, (**C**) a no significant effect of rapamycin on Pink1 immunofluorescence at any time interval, and (**D**) a significant increase in the number of puncta positive for both PARK and Pink1, which exceeds at large the increase induced in PARK immunofluorescence alone. Data are given as the mean±S.E.M. from N=100 cells per group. *$p<0.05$ c ompared with c ontrols. S cale ba r= 20 µ m.

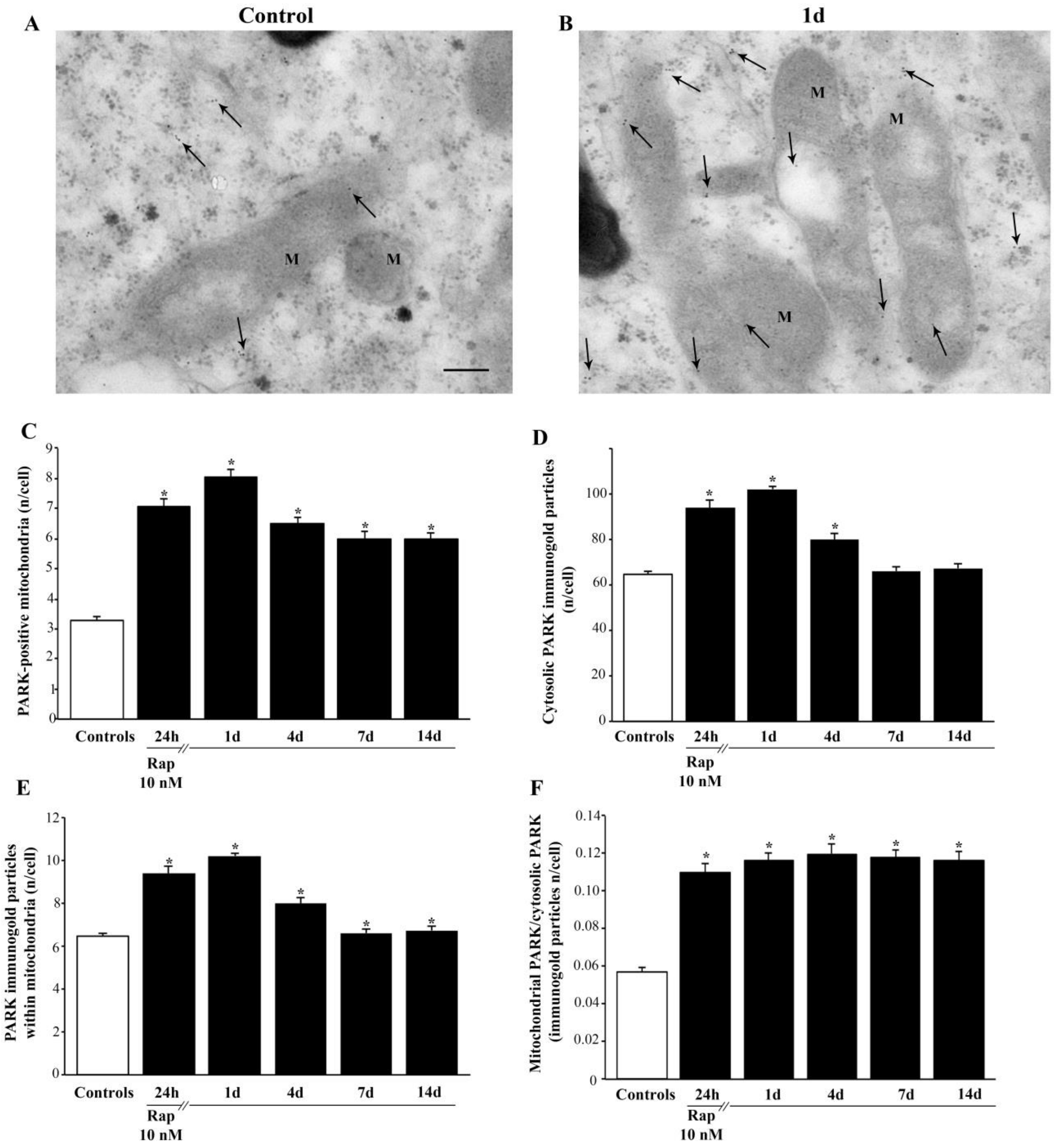

Figure S20. Rapamycin increases PARK immuno-gold particles in A172 cells. Representative micrographs showing PARK immuno-gold in A172 cells of control (**A**) and after 1d of rapamycin withdrawal (**B**). Graphs report: (**C**) PARK-positive mitochondria, (**D**) PARK immuno-gold particles within the cytosol, (**E**) PARK immuno-gold particles within mitochondria, and (**F**) the ratio of mitochondrial to cytosolic PARK particles. Values are given as the mean±S.E.M. from N=50 cells per group. *p<0.05 compared with controls. Scale bar=140 nm.

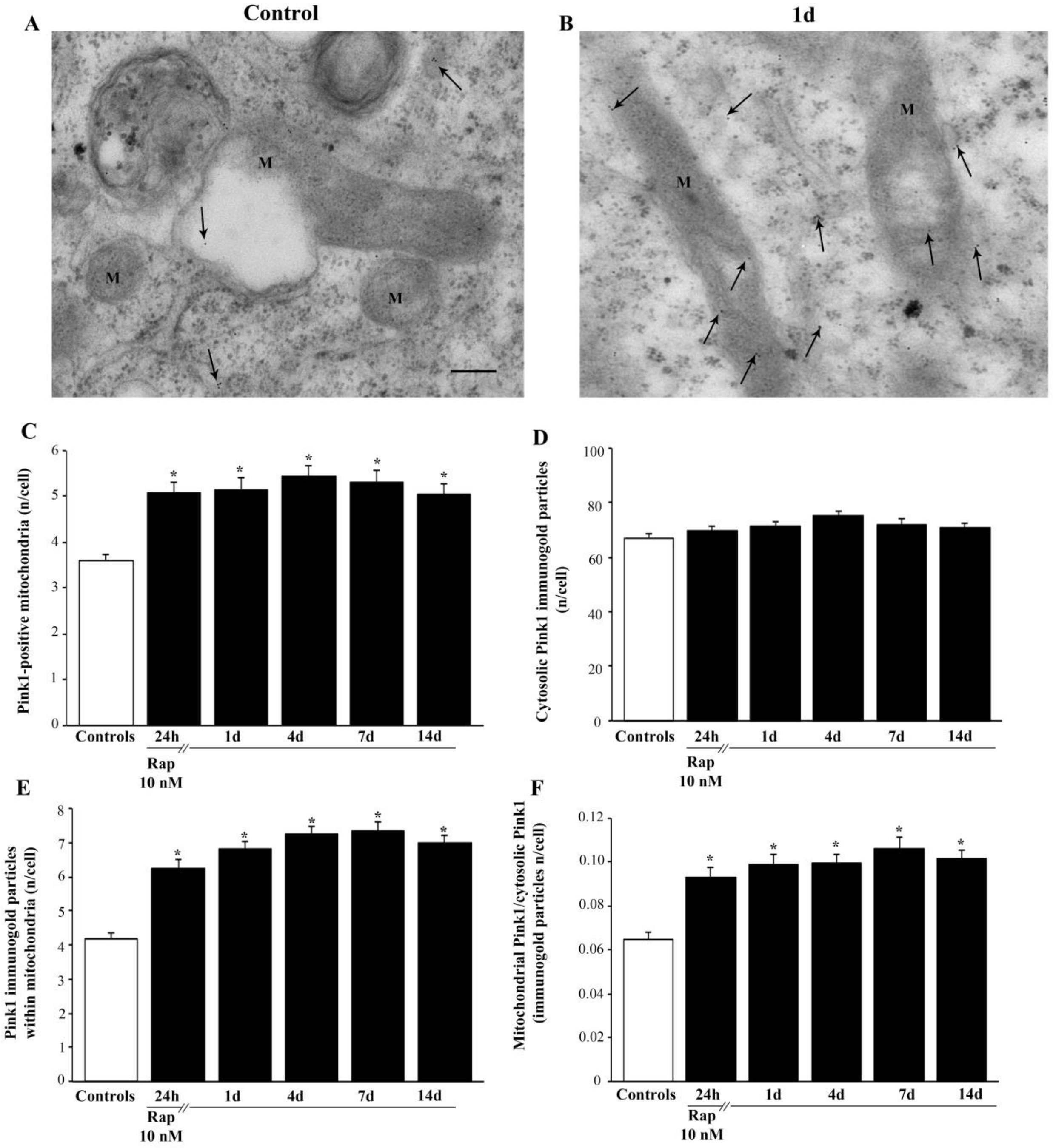

Figure S21. Rapamycin increases Pink1 immuno-gold particles within mitochondria of A172 cells. Representative micrographs showing Pink1 immuno-gold in A172 cells of control (**A**) and after 1d of rapamycin withdrawal (**B**). Graphs report (**C**) Pink1-positive mitochondria, (**D**) Pink1 immuno-gold particles within the cytosol, (**E**) Pink1 immuno-gold particles within mitochondria, and (**F**) the ratio of mitochondrial to cytosolic Pink1 particles. Values are given as the mean±S.E.M. from N=50 cells per group. *$p<0.05$ compared with controls. Scale bar=140 nm.

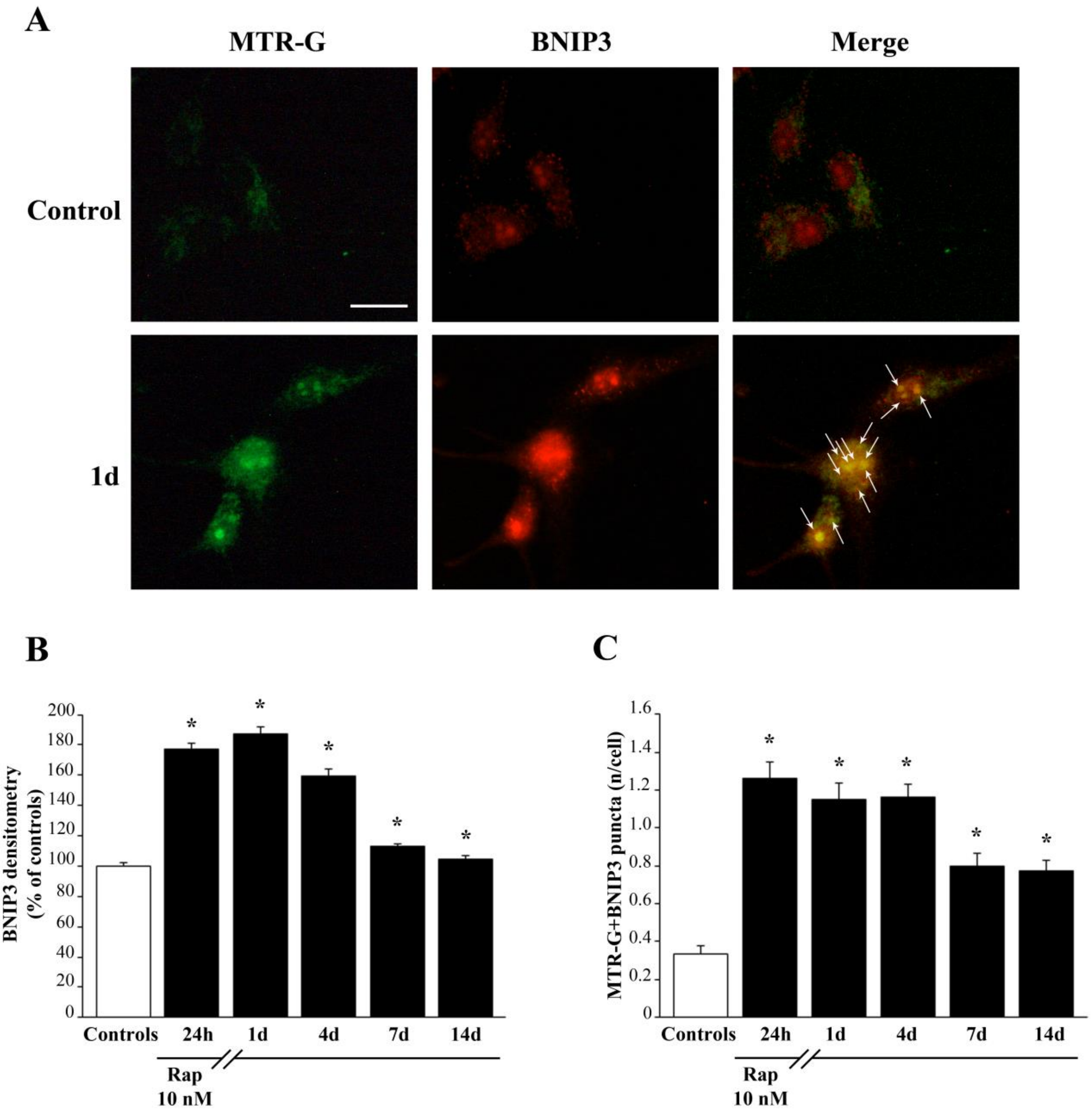

Figure S22. Rapamycin increases the merging of MTR-G with BNIP3 in A172 cells. (**A**) Representative pictures showing that rapamycin increases MTR-G fluorescence along with LC3 immunofluorescence compared with control. Arrows point to merging (yellow) puncta. (**B**) Graph reports the rapamycin-induced long-lasting increase in BNIP3 immunofluorescence. (**C**) Graph reports the rapamycin-induced long-lasting increase in MTR-G+BNIP3 merging puncta. Data are given as the mean±S.E.M. from N=100 cells per group. *$p<0.05$ compared with controls. Scale bar=30 μm.

A

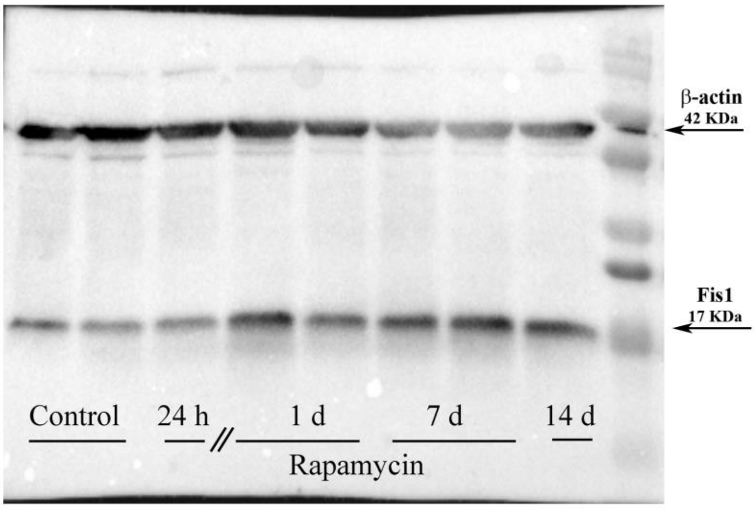

B

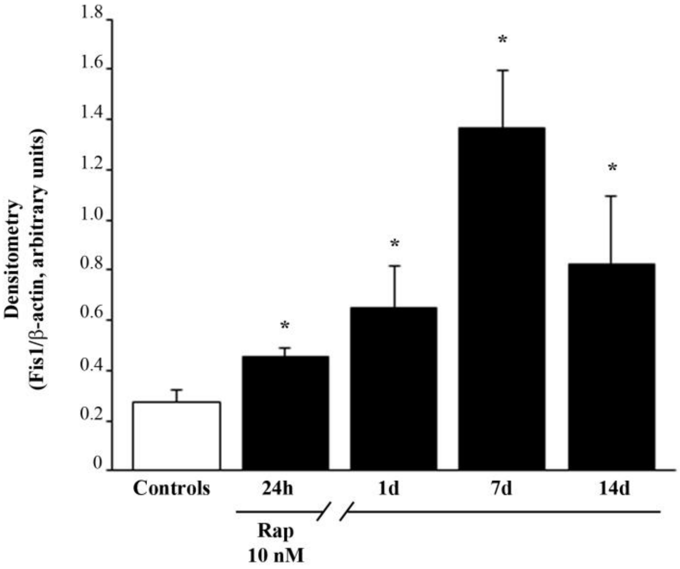

Figure S23. Rapamycin increases the fission protein marker Fis1. (**A**) Representative western blotting of Fis1 protein. (**B**) Graph reports the rapamycin-induced increase of the levels of Fis1 at all time intervals. Values are given as the mean±S.E.M. from at least 4 samples per group. *$p \leq 0.05$ compared with controls.

A

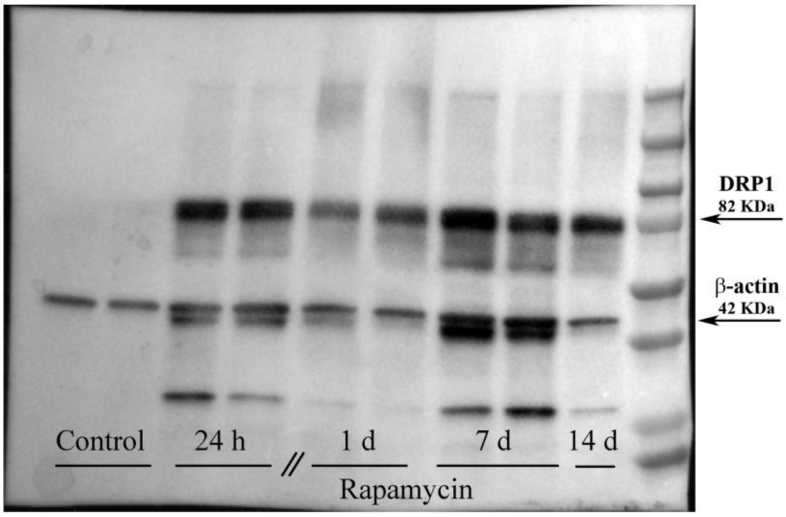

B

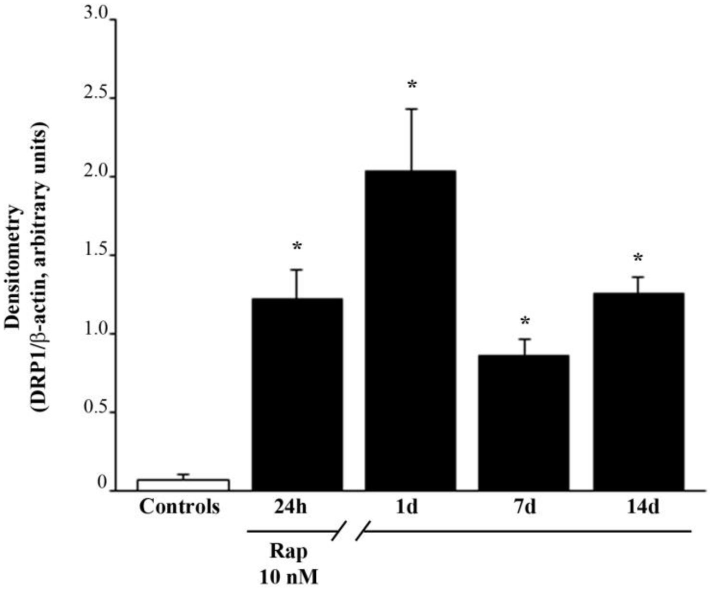

Figure S24. Rapamycin increases the fission protein marker DRP1. (**A**) Representative western blotting of DRP1 protein. (**B**) Graph reports the rapamycin-induced increase of the levels of DRP1 at all time intervals. Values are given as the mean±S.E.M. from at least 3 samples per group. *$p \leq 0.05$ compared with controls.

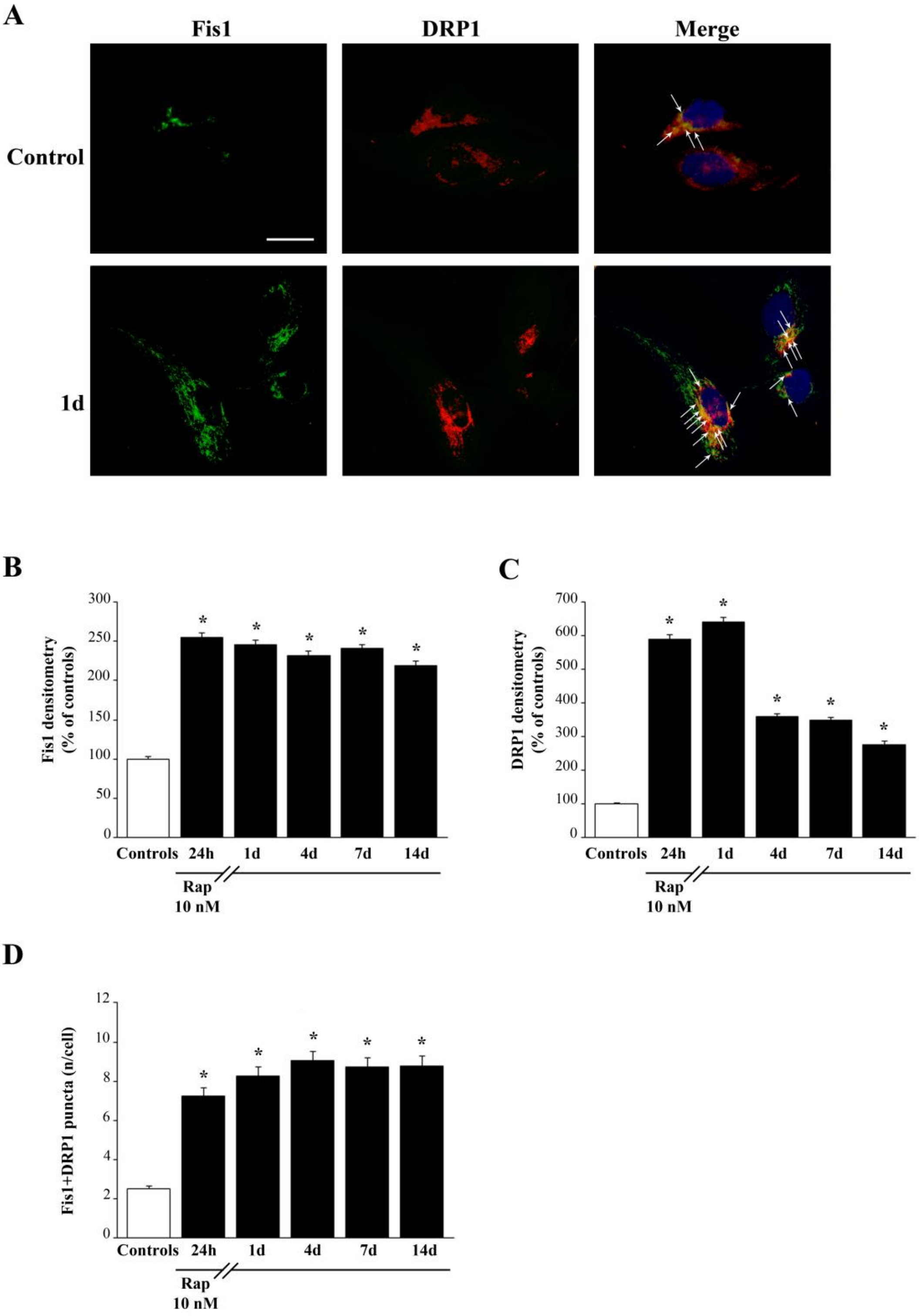

Figure S25. Rapamycin increases immunofluorescence for Fis1 and DRP1 in A172 cells. (**A**) Representative pictures of the fission markers Fis1 and DRP1. Arrows point to merging (yellow) puncta. Graphs report (**B**) Fis1 and (**C**) DRP1 immunofluorescent intensity, while graph (**D**) reports Fis1+DRP1 merging puncta. Values are given as the mean±S.E.M from N=100 cells per group. *$p<0.05$ compared with controls. Scale bar=16 μm.
